# New *de novo* assembly of the Atlantic bottlenose dolphin (*Tursiops truncatus*) improves genome completeness and provides haplotype phasing

**DOI:** 10.1093/gigascience/giy168

**Published:** 2019-01-29

**Authors:** Karine A Martinez-Viaud, Cindy Taylor Lawley, Milmer Martinez Vergara, Gil Ben-Zvi, Tammy Biniashvili, Kobi Baruch, Judy St. Leger, Jennie Le, Aparna Natarajan, Marlem Rivera, Marbie Guillergan, Erich Jaeger, Brian Steffy, Aleksey Zimin

**Affiliations:** 1 Illumina, Inc., 5200 Illumina Way, San Diego, CA 92122, USA; 2 GinkgoFish LLC, 204 West Spear St, Carson City, NV 89703; 3 Plant With Purpose, 4747 Morena Blvd, San Diego, CA 92117, USA; 4 NRGene, 5 Golda Meir St., Ness-Ziona 7403649, Israel; 5 SeaWorld San Diego, 500 Sea World Dr., San Diego, CA 92109, USA; 6 Ocean Discovery Institute, 4255 Thorn St., San Diego, CA 92105 USA; 7 Johns Hopkins University, Welch Library of Medicine, Ste 105, 1900 E. Monument St., Baltimore, MD 21205, USA

**Keywords:** *de novo* genome assembly, bottlenose dolphin, *Tursiops truncatus*, 10x Genomics, DeNovoMAGIC, Illumina

## Abstract

High-quality genomes are essential to resolve challenges in breeding, comparative biology, medicine, and conservation planning. New library preparation techniques along with better assembly algorithms result in continued improvements in assemblies for non-model organisms, moving them toward reference-quality genomes. We report on the latest genome assembly of the Atlantic bottlenose dolphin, leveraging Illumina sequencing data coupled with a combination of several library preparation techniques. These include Linked-Reads (Chromium, 10x Genomics), mate pairs (MP), long insert paired ends, and standard paired end. Data were assembled with the commercial DeNovoMAGIC assembly software, resulting in two assemblies, a traditional “haploid” assembly (Tur_tru_Illumina_hap_v1) that is a mosaic of the two parental haplotypes and a phased assembly (Tur_tru_Illumina_phased_v1) where each scaffold has sequence from a single homologous chromosome. We show that Tur_tru_Illumina_hap_v1 is more complete and more accurate compared to the current best reference based on the amount and composition of sequence, the consistency of the MP alignments to the assembled scaffolds, and on the analysis of conserved single-copy mammalian orthologs. The phased *de novo* assembly Tur_tru_Illumina_phased_v1 is the first publicly available for this species and provides the community with novel and accurate ways to explore the heterozygous nature of the dolphin genome.

## Introduction

Technical advances in the past decade have reduced sequencing costs and improved access to sequencing data. Subsequent improvements in DNA extraction, preparation, and assembly algorithms facilitate low-cost accurate *de novo* genome assemblies. Such assemblies are essential for constructing haplotype diversity databases for breeding, comparative biology, medicine, and conservation planning. Even highly complex genomes now benefit from higher contiguity and improved protein coding coverage [1–4]. Consortium efforts to catalogue biodiversity of pivotal species of comparative evolutionary significance will continue to drive novel low-cost approaches toward reference-quality assemblies with chromosome-level resolution [5–7]. Here, we use a combination of methods to drive improvements in assembly structure for the Atlantic bottlenose dolphin (*Tursiops truncatus*; National Center for Biotechnology Information [NCBI]: txid9739). This genome assembly, like those of the Hawaiian Monk seal and African wild dog, is being published with the goal to facilitate research on comparative genomics, provide structure for cataloging biodiversity, and ultimately support decisions around species conservation and management [8, 9].

The bottlenose dolphin is one of the most widely studied marine mammals; however, the taxonomy of the *Tursiops* genus remains unresolved. Numerous species designations have been suggested but not adopted due to a lack of resolution afforded by available data [10]. Even with new molecular genetic markers, we have reached a limitation on resolution from genetic data available to delineate species, subspecies, and populations [11]. To usher this species into the era of genomics, a high-quality reference genome is essential. It provides structure to catalogue diversity within and between species at the whole-genome level. In addition, the parallel molecular trajectory between dolphin and other mammalian species [12] makes the bottlenose dolphin a useful model to understand aspects of human health such as metabolic processes/diabetes [13–15], proteomics [16, 17], and aging [18].

A preliminary dolphin genome was first submitted to NCBI ( Turtru1.0; GCA_000151865.1) using low-coverage (2.82X) Sanger sequencing for the purpose of cross-species comparison [12, 19, 20]. Subsequent improvements were achieved through the addition of 30X Illumina short read data and 3.5 × 454 data (Ttru_1.4; GCA_000151865.3). A much more complete genome was submitted in 2016 leveraging improvements in library preparation and assembly methods (Meraculous v. 2.2.2.5 and HiRise v. 1.3.0–116-gf50c3ce; Dovetail, Inc) with 114X coverage of Illumina HiSeq data prepared using a proximity ligation Hi-C protocol (Tur_tru v1; GCA_001922835.1; [16]).

With the collection of data from multiple sources including Linked-Reads (Chromium, 10x Genomics; [21]), mate pairs (MP), long insert paired ends, and standard paired ends, and using DeNovoMAGIC assembly tool (NRGene), we provide an improved haploid reference-quality dolphin genome assembly as well as the first haplotype phased diploid assembly. We refer to our unphased assembly as Tur_tru_Illumina_hap_v1 and to the phased assembly as Tur_tru_Illumina_phased_v1. Using Tur_tru v1 for comparison, our assembly shows increased contiguity and completeness with high consistency to the MP data and orthologous mammalian protein alignments. Additionally, by aligning Tur_tru_Illumina_hap_v1 to the human reference genome, we illustrate the synteny of the dolphin scaffolds to human chromosome 1 [22, 23].

## Results

### Coverage

We generated sequence data for a total coverage of approximately 450X, the majority from PCR Free and Chromium 10X Genomics Linked-Read libraries (Table 1). Coverage was computed using a 2.4 Gbp estimated genome size. Genome assembly was conducted using DeNovoMAGIC software (NRGene). More detail about the library preparation and the assembly process are found in the Methods section.

**Figure 1:**
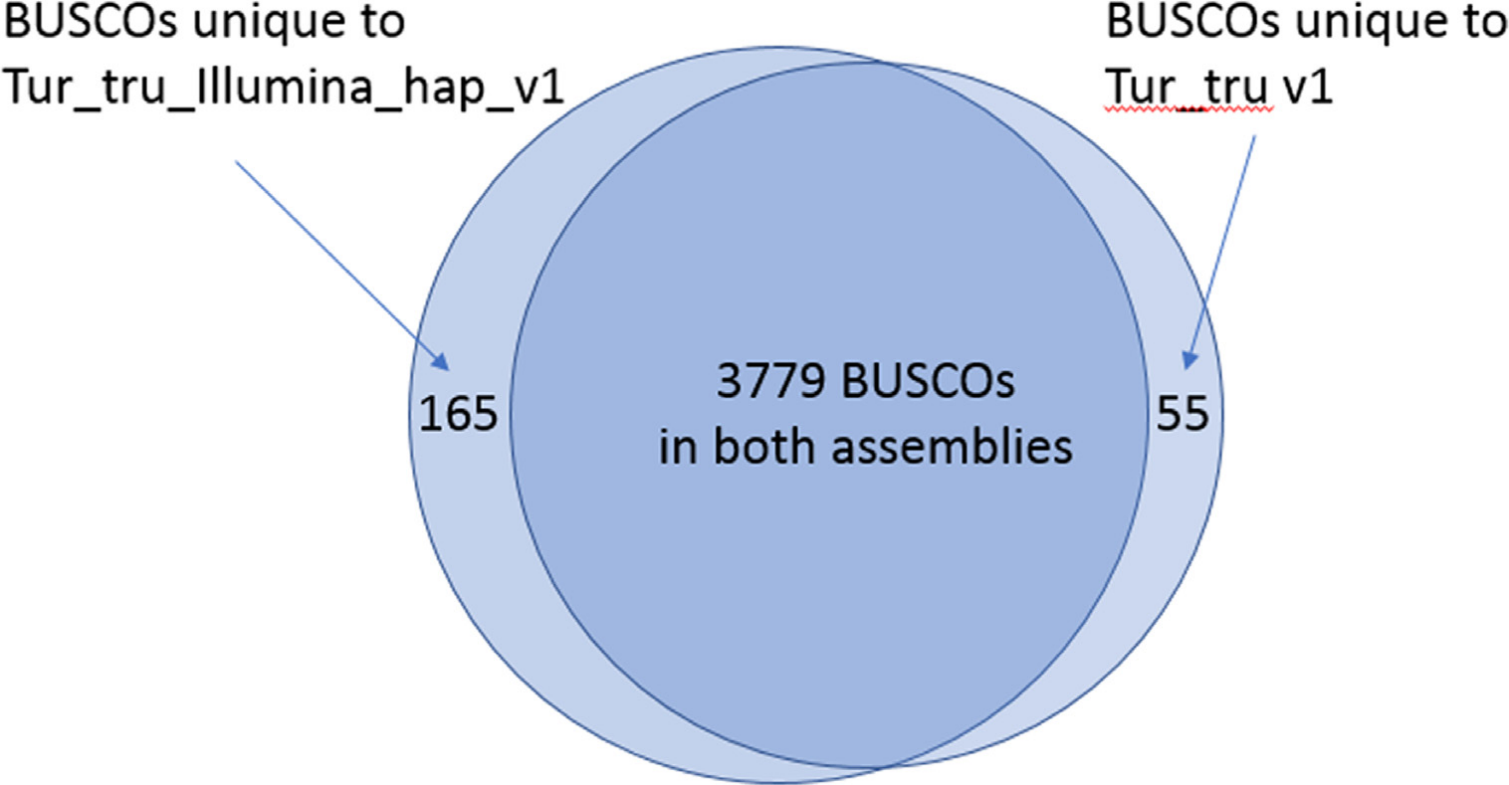
Venn diagram of BUSCOs present in two dolphin assemblies. Out of 4,104 BUSCOs in the mammalia set, 105 are missing from both assemblies. Our assembly has 165 BUSCOs not present in Tur_tru v1, and Tur_tru v1 has 55 BUSCOs that are not present in our assembly.

**Table 1:** Summary of the sequencing data collected to create Tur_tru_Illumina_hap_v1 and Tur_tru_Illumina_phased_v1

Library type	Read length	Insert size	Genomic coverage
**PCR-free**	2 × 250 bp	450 bp	101x
**PCR-free**	2 × 160 bp	800 bp	123x
**Mate-Pair**	2 × 150 bp	2–4 Kbp (peak 4.2 Kbp)	37x
**Mate-Pair**	2 × 150 bp	5–7 Kbp (peak 6.0 Kbp)	61x
**Mate-Pair**	2 × 150 bp	8–10 Kbp (peak 9.9 Kbp)	58x
**10X Chromium**	2 × 150 bp	–	70x

### Haploid and diploid assemblies

Here, we report on two assemblies, one traditional haploid consensus assembly Tur_tru_Illumina_hap_v1 that represents a mosaic of the maternal and paternal haplotypes and the other haplotype-phased (i.e., diploid) assembly where each scaffold represents sequence corresponding to a single haplotype, Tur_tru_Illumina_phased_v1. The quantitative statistics for both assemblies are listed in Table 2. The phased or diploid genome assembly, which was made possible using Illumina sequencing data by leveraging the combination of library prep methods including Linked-Reads, is a significant advance and will provide the community with a powerful genomic tool for the downstream analysis in the context of the true heterozygous dolphin genome.

**Table 2:** Comparison of quantitative statistics for different assemblies of the bottlenose dolphin

	Tur_tru v1	Tur_tru_Illumina_hap_v1	Tur_tru_Illumina_phased_v1
**Total sequence**	2,120,283,832	2,383,130,043	4,678,362,582
**No. of scaffolds**	2,647	481	98,209
**Longest scaffold**	96,299,184	83,924,496	10,429,594
**Scaffold N50**	23,564,561	26,997,441	777,432
**Scaffold L50**	26	30	1,509
No.**of contigs**	116,650	139,544	355,974
**Longest contig**	403,070	320,783	298,006
**Contig N50**	37,749	30,985	25,997
**Contig L50**	17,321	23,199	53,738
**GC content**	40.85	41.25	41.95

The total sequence listed excludes Ns (ambiguous nucleotides). Ns were also squeezed out from the scaffolds for N50 computations. We used genome size of 2,383,130,043 bp, equal to the total amount of sequence in the scaffolds of the bigger haploid assembly, for comparison of the N50 contig and scaffold sizes between the two assemblies. The Tur_tru_Illumina_hap_v1 and Tur_tru v1 assemblies have comparable scaffold N50 sizes, and Tur_tru v1 has bigger contigs. The Tur_tru_Illumina_hap_v1 assembly has more sequence and our Benchmarking Universal Single-Copy Orthologs (BUSCO) analysis (Table 3) shows that it is likely more complete. The N50 comparisons to the haplotype-resolved Tur_tru_Illumina_phased_v1 assembly are shown for completeness, computed with 2x genome size (2*2,383,130,043 = 4,766,260,086 bp).

### Genome assembly comparison

Both assemblies were compared to the best available assembly Tur_tru v1 (NCBI accession GCA_001922835.1; [16]). We did not use the Ttru_1.4 assembly (NCBI accession GCA_000151865.3) because the contiguity statistics of the Ttru_1.4 are vastly inferior to the Tur_tru v1 with a contig N50 3 times smaller than Tur_tru v1 and scaffold N50 over 200 times smaller.

The statistics for the Tur_tru_Illumina_hap_v1 assembly show bigger scaffolds but slightly smaller contigs, with about 13% more sequence in the scaffolds compared to Tur_tru v1 (Table 2). More sequence does not necessarily make for a better assembly considering that the extra sequence may be duplicated haplotypes or contaminants that do not belong to the original organism. To characterize the extra sequence, we first aligned the Tur_tru_Illumina_hap_v1 to the Tur_tru v1 assembly using the Nucmer aligner, which is part of MUMmer4 package [24]. We used default settings for generating the alignments. We then analyzed the alignments using the dnadiff package included with MUMmer4. We found that 87.5% of Tur_tru_Illumina_hap_v1 sequence aligned to 97.9% of Tur_tru v1. This shows that 12.5% of Tur_tru_Illumina_hap_v1 had no alignments to Tur_tru v1, while only 2.1% of Tur_tru v1 had no alignments to Tur_tru_Illumina_hap_v1. Therefore, there are 301 Mbp of extra novel sequence in our new assembly Tur_tru_Illumina_hap_v1. We then used the Benchmarking Universal Single-Copy Orthologs (BUSCO) (BUSCO, RRID:SCR_015008) tool to show that the extra sequence is meaningful (Table 3). Tur_tru_Illumina_hap_v1 had 160 missing BUSCOs compared to 270 missing in Tur_tru v1. The number of duplicated BUSCOs was higher by only 34 in our assembly compared to Tur_tru v1. This suggests that most of the extra sequence in Tur_tru_Illumina_hap_v1 is not contamination or redundant sequence and likely contains useful coding information. There were 105 BUSCOS missing from both assemblies. We examined the locations of the 165 BUSCOs that are only found in the Tur_tru_Illumina_hap_v1, and all of them fully or partially aligned to locations in the sequences that were missing in Tur_tru v1 assembly. Figure 1 shows the Venn diagram of BUSCOs aligned to both assemblies, showing that there are 165 BUSCOS that are only present in Tur_tru_Illumina_hap_v1 and 55 that are only present in Tur_tru v1, with 3,779 present in both assemblies. The haplotype-resolved assembly is more fragmented, and it is missing 266 BUSCOs. As expected, most of the complete BUSCOs that were found (3537) are duplicated 2227, since they are found in different haplotypes.

**Table 3:** Comparison of BUSCO 3.0.2 Mammalia single copy orthologs among the three Dolphin assemblies

BUSCOs	Tur_tru v1	Tur_tru_Illumina_hap_v1	Tur_tru_Illumina_phased_v1
Complete	3,647	3,837	3,537
Complete single-copy	3,614	3,760	1,310
Complete duplicated	33	77	2,227
Fragmented	187	107	301
Missing	270	160	266
Total	4,104	4,104	4,104

The table shows that the Tur_tru_Illumina_hap_v1 assembly is more complete, with 110 fewer missing single-copy orthologs compared to the Tru_tru v1 assembly. The Tur_tru_Illumina_hap_v1 assembly has 43 extra duplicated orthologs, which possibly points to incomplete filtering of redundant haplotypes. While the Tur_tru v1 assembly has bigger contigs, the Tur_tru_Illumina_hap_v1 assembly has many fewer fragmented BUSCOs. The haplotype-resolved Tur_tru_Illumina_phased_v1 assembly is less contiguous and less complete. As expected, more than half of the complete BUSCOs are duplicated, corresponding to the two resolved haplotypes.

### Assembly validation through MP consistency

Since both Tur_tru v1 and Tur_tru_Illumina_hap_v1 reference the same species, we expect few rearrangements between the assemblies. To examine this, we compared the absolute and relative correctness of the scaffolds of Tur_tru_Illumina_hap_v1 assembly by aligning the Illumina data from the 5–7 Kbp MP library to the scaffolds of Tur_tru_Illumina_hap_v1, Tur_tru_Illumina_phased_v1, and Tur_tru v1 assemblies using the Bowtie2 (Bowtie 2, RRID:SCR_016368) tool [25]. We chose this library because it contained the largest number of valid 5–7 Kbp MPs. We then used only high-quality uniquely aligning mated reads (both mates had to align uniquely with quality score 42 in the SAM file) and classified the alignments of the MPs into the following categories (Table 4):

1. **Same scaffold happy**—number of MPs where both mates aligned to the same scaffold in the correct orientation with mate separation within 3 standard deviations of the library mean.
2. **Same scaffold short**—number of MPs where both mates aligned to the same scaffold in the opposite orientation with mate separation of less than 1,000 bp; these MPs are not indicative of scaffolding misassemblies, they are simply a by-product of the MP library preparation process as they are MPs that are missing the circularization junction site between the mates.
3. **Same scaffold long**—number of MPs where both mates aligned to the same scaffold in the correct orientation, but the mate separation exceeded 3 standard deviations of the library mean.
4. **Same scaffold misoriented—**number of MPs where both mates aligned to the same scaffold in the opposite orientation with mate separation of more than 1,000 bp.
5. **Mates aligned to different scaffolds**—number of MPs where the two mates aligned to different scaffolds.
6. **Only one mate in the pair aligned**—number of MPs where only one read aligned to the assembly.

**Table 4:** Comparison of the number of MPs from 5–7 Kbp library uniquely aligned to Tur_tru_Illumina_hap_v1, Tur_tru_Illumina_phased_v1, and Tur_tru v1 assemblies

	Tur_tru_Illumina_hap_v1	Tur_tru_Illumina_phased_v1	Tur_tru v1
**Same scaffold mate ALL**	228,285,253	73,514,546	219,277,963
**Same scaffold happy**	125,224,663	37,248,420	117,942,390
**Same scaffold misoriented**	158,547	51,533	1,164,118
**Same scaffold long**	82,948	9,183	274,458
**Same scaffold short**	102,819,095	36,205,410	99,896,997
**Mates aligned to different scaffolds**	5,629,393	7,38,471	5,576,802
**One mate in the pair aligned**	61,284,891	26,228,913	68,707,025

The alignments were done with Bowtie2. Only the reads that mapped *uniquely* were used for this computation, thus the number of MPs uniquely mapping to haplotype resolved assembly is much smaller. Same scaffold means that both mates mapped to the same scaffold; happy mates aligned in the correct orientation with mate distance within 5 standard deviations from the mean; misoriented mates aligned in the wrong orientation; long mates aligned with the distance between the mates exceeding 5 standard deviations; short mates aligned with the distance of less than 1,000 bp. Same scaffold mate ALL is the total number of all MPs where both mates aligned to the same scaffold.

The “Same scaffold mate ALL” category in Table 4 is the sum of all mates in categories 1 to 4 (happy, short, long, and misoriented); it is listed for completeness.

Comparing Tur_tru_Illumina_hap_v1 with Tur_tru v1, the total number of reads uniquely aligning to both “haploid” assemblies is similar; 295.2 M reads aligned to Tur_tru_Illumina_hap_v1 vs 293.6 M reads aligned to Tur_tru v1. The total number of MPs aligning to the same scaffold is larger for Tur_tru_Illumina_hap_v1. Of the MPs aligning to the same scaffold, the number of MPs in the ”Same scaffold happy” category is similar between the two assemblies. The differences that stand out are the much larger (7.3 times more) number of mates that aligned to the same scaffold in the wrong orientation and the much larger (3.3 times more) number of the same scaffold long pairs in Tur_tru v1 compared to Tur_tru_Illumina_hap_v1 (Table 4). Of course, some level of discrepancy is expected because the two assemblies represent two different individuals with an unknown level of structural variation between them. However, in concert, the two different categories may also suggest a possibility of a relatively higher number of locally mis-ordered or mis-oriented contigs in the scaffolds of the Tur_tru v1 assembly. This may be due to the scaffolding process used to create the Tur_tru v1 assembly. The assembly was created with the HiRise assembler [26] using proximity ligation Hi-C data for scaffolding. The proximity ligation data provide MPs of all possible sizes; however, the MP distances and mate orientations are unknown. Since there are more shorter pairs than longer pairs due to the 3D structure of the DNA, it is much more likely to ligate parts of DNA that are closer to each other than the ones that are far apart. This property enables one to use these data for scaffolding. By mapping the pairs to the assembled scaffolds, one can measure how the distance between the mates in a pair varies with the number of pairs whose ends map to the same location in the assembly. However, the dependence is weak on the short end, meaning that the number of pairs of about 10 Kbp in length is not much different from the number of links of 12–13 Kbp in length. This frequently results in mis-orientations and shuffling of scaffold positions for contigs or scaffolds that are smaller than 10–20 Kbp in the scaffolding process.

The haplotype phased assembly is much more fragmented compared to both haploid assemblies, resulting in higher relative numbers of MPs mapping to different scaffolds. However, when looking at the “internal” MPs, i.e., where both mates map at least 10 Kb away from the scaffold ends, we see remarkable consistency, with less than 0.5% of the mates mapped to the wrong scaffold (see next section). Since for this analysis we only used mates mapping uniquely to the assembly, and there are two copies of the genome in the assembly, the total number of mapped mates is much lower.

### Haplotype resolution

**Figure 2:**
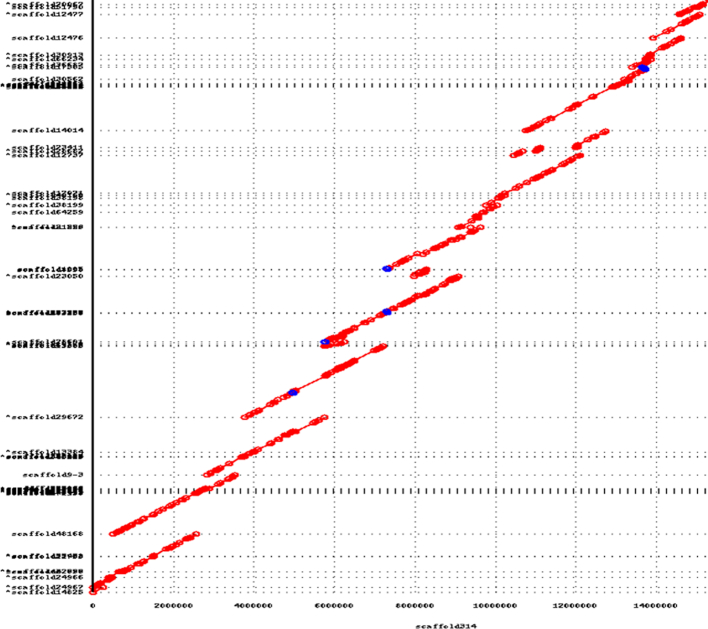
An example mummerplot of the alignments of the phased assembly to the “haploid” one, spanning about 15 Mbp of sequence of scaffold314. The circles represent contig ends, with lines joining them representing aligned sequence. The color indicates the direction of the alignment, with red and blue forward and reverse, respectively. We show that for most locations on the *x*-axis (haploid assembly coordinates) there are two alignments on the *y*-axis corresponding to the two phased haplotypes. The small number of regions with a single contig aligning represent long homozygous regions of the genome that we were unable to phase.

To Illustrate the resolution of the haplotypes in Tur_tru_Illumina_phased_v1, we aligned it to Tur_tru_Illumina_hap_v1 using the Nucmer tool. In Fig. 2 we show the mummerplot of alignments of the phased assembly to the haploid one. The circles represent contig ends, with lines joining them representing aligned sequence. The color indicates direction of the alignments. We display the alignments to an arbitrarily chosen scaffold314 of the haploid assembly. Only alignments longer than 5 Kb are shown. Figure 2 shows that most of the “haploid” assembly aligns to two phased scaffolds, i.e., for each location on the *x*-axis there are two corresponding alignments on the *y*-axis. The regions that are covered by a single haplotype (rather than 2) are most probably homozygous regions of this genome. In cases where the homozygous region is long, it is more difficult to phase its heterozygous ends. Thus, in some cases, the homozygous regions are represented only once in the phased assembly (instead of twice). This is the cause for some of the single copy BUSCOs in the phased assembly. In our experience, this issue is more pronounced in mammalian phased assemblies due to the relatively lower heterozygosity level and the way it is distributed along the genome.

In haplotype phasing, it is easy to phase small regions. For example, a single isolated single-nucleotide polymorphism (SNP) with no haplotype differences within 100 bp in both directions can be trivially phased into two 201 bp (or longer) contigs different by one base in the middle. It gets more difficult for larger contigs/scaffolds, where one must make sure that the contig/scaffold represents single haplotype and not a “mosaic” of haplotypes and that the SNPs and other bigger haplotype differences are correctly “phased.” To do that, we mapped the MPs from the 5–7 Kb MP library to all phased scaffolds using Bowtie2 [25] and then examined the “internal” MPs where both reads in each pair mapped to the assembly and one read mapped within 10 Kb away from the ends of the scaffold. This would imply that if haplotype phasing is done properly, the other mate must map to the same scaffold and not its haplotype. If it does not, then it indicates an apparent mis-assembly or failure to phase haplotypes. By measuring the number of “properly” aligned internal mates, where both mates aligned to the same scaffold vs “improper” internal mates where the mates aligned to different scaffolds, one can measure the efficacy of the haplotype phasing. There were 35,697,369 pairs where both mates mapped properly to the same scaffold, while only 169,244 mapped improperly, i.e., to two different scaffolds. The percentage of improperly mapping MPs is only 0.5%, indicating that haplotype resolution was of high quality.

### Synteny between human and dolphin

**Figure 3:**
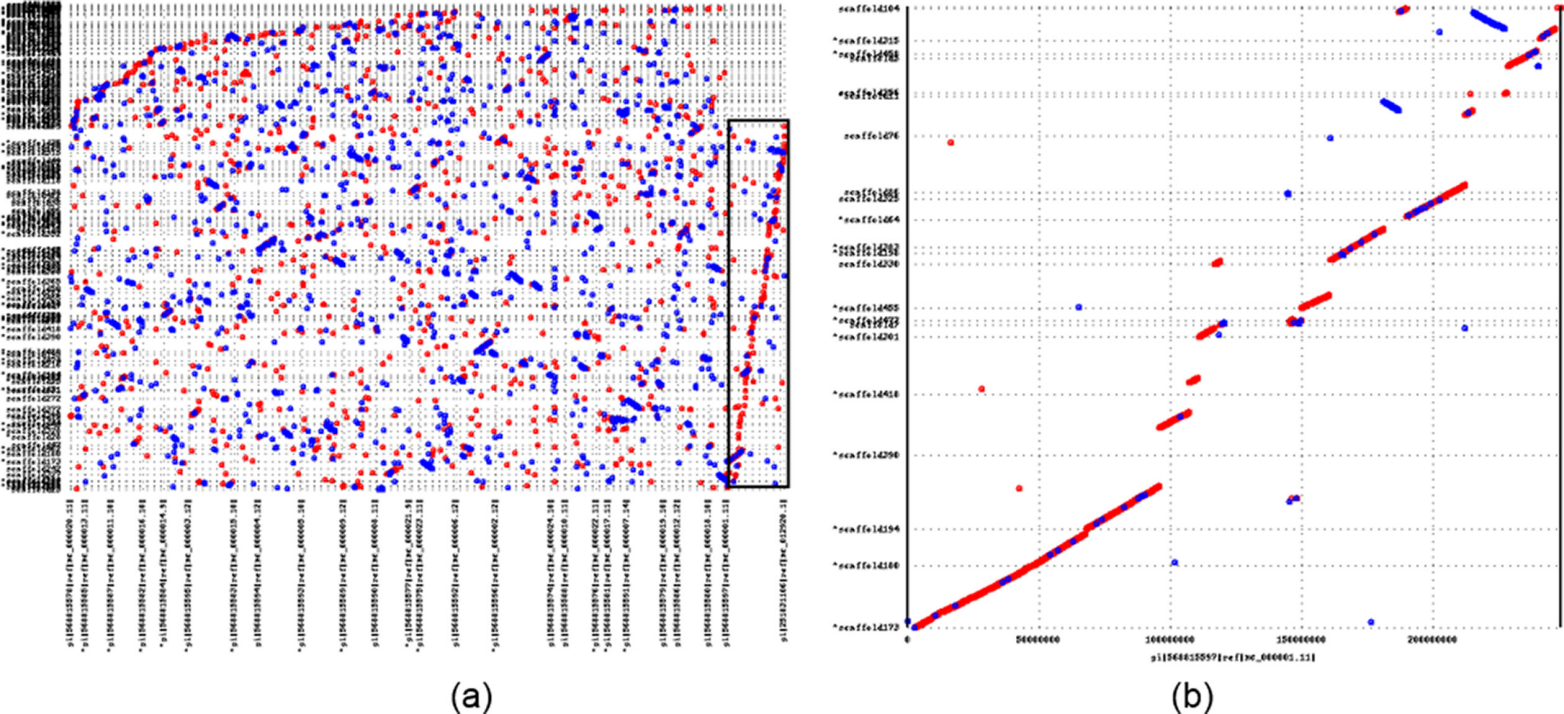
This figure shows the alignment of the Tur_tru_Illumina_hap_v1 assembly to the human GRCH38 reference (primary chromosomes only). Each dot represents an alignment, with red indicating forward direction and blue indicating reverse direction. Human reference coordinates are on the *x*-axis and Tur_tru_Illumina_hap_v1 assembly alignment coordinates are on the *y*-axis. **(A)** Alignment of the entire assembly to the human reference with alignments to human chromosome 1 highlighted by the black box. One can clearly see the synteny that is present between the dolphin scaffolds and human chromosome 1. No other human chromosome shows clear synteny. Dolphin scaffolds with syntenic alignments spanning over 50% of the scaffold were extracted. **(B)**Alignments only to human chromosome 1.

Dolphin is a mammal, and currently the best mammalian reference genome is the human genome. To understand similarities between dolphin and human on the DNA level, we aligned the Tur_tru_Illumina_hap_v1 assembly to the primary chromosomes of the current haploid human reference genome GRCh38 [27]. Since human and dolphin are fairly distant species, we did not expect to find long DNA sequence alignments; instead, we were looking for synteny where relatively short DNA fragments of scaffolds align in the same order and orientation between the two assemblies. We used the MUMmer4 package for producing the alignments using the default settings. The alignment mummerplot (Fig. 3) shows a striking synteny between the dolphin assembled scaffolds and human chromosome 1, visible even on the large-scale chromosome plot (Fig. 3a). No large-scale synteny to the other human chromosomes can be readily observed. The synteny observation is possible due to large scaffold sizes in Tur_tru_Illumina_hap_v1. In Fig. 3b, we show 22 scaffolds that have 50% or more of their sequence in syntenic alignments. The syntenic alignments of these 22 scaffolds span nearly the entire human chromosome 1 sequence. The synteny is not a new finding, it was first identified by Bielec et al [22] and was later extended to many other placental mammals [23]. The Tur_tru_Illumina_hap_v1 assembly clearly illustrates and confirms the expected synteny.

## Methods

### Sample collection and DNA extraction

The sample for this study came from a female Atlantic bottlenose dolphin (sample ID 04329), captive born at SeaWorld of Orlando, Florida, from wild male and female Atlantic bottlenose dolphins. The animal was 36 years old at blood collection with a healthy medical history. Blood was collected using PAXgene Blood DNA Tubes (Qiagen). High-molecular-weight genomic DNA was isolated using the MasterPure DNA Purification Kit (Illumina) and subsequently quantified and qualified using Quant-iT dsDNA Kit and E-Gel EX Agarose Gel (ThermoFisher).

### Collecting sequence data

#### Paired end libraries

We generated the 450 bp and 800 bp paired-end (PE) libraries using the TruSeq PCR-free DNA Sample Prep kit (Illumina). The protocol was slightly modified at fragmentation and double-size selection steps by adjusting the DNA shearing protocols (Covaris) and by empirically titrating the ratios of SPRI magnetic beads over DNA to obtain insert sizes around 450 bp and 800 bp. We then evaluated the libraries for insert size and yield using Bioanalyzer (Agilent) and real-time qPCR assay, using Illumina DNA Standards and primer master mix qPCR kit (KAPA Biosystems, Roche), then normalized to 2 nM prior to clustering and sequencing. Both the 450 bp and 800 bp libraries were then denatured and diluted to 8 pM and 12 pM, respectively. The 800 bp PE library was clustered and sequenced on the HiSeq 2000, using the HiSeq Cluster and SBS v4 kits for PE 2 × 160 bp reads (Illumina). The 450 bp PE library was clustered and sequenced on the HiSeq 2500 v2 Rapid Run mode using the HiSeq Rapid Cluster and SBS v2 kits for PE 2 × 250 bp reads.

#### Mate pair libraries

To maximize sequence diversity and genome coverage, three separate MP libraries were constructed corresponding to 2–5 Kb, 5–7 Kb, and 7–10 Kb insert sizes using the Nextera MP Library Preparation Kit according to the manufacturer's instructions (Illumina). All three libraries were generated from a single input of 4ug of genomic DNA size-selected on a 0.8% E-gel (Invitrogen). Proper sizing of gel-extracted products was confirmed using the Bioanalyzer High Sensitivity chip (Agilent), and 600 ng was subsequently used as input for circularization. Following library preparation, the Bioanalyzer was used to confirm library quality. Each of the three libraries were quantified by qPCR (KAPA Biosystems Library Quantification Kit, Roche), denatured, and diluted to 200 pM after size-adjustment, according to Bioanalyzer results, and clustered on the cBot according to the manufacturer's instructions (Illumina). Then, 2 × 150 bp of Illumina PE sequencing was performed on the HiSeq 4000 using the HiSeq 3000/4000 Cluster and SBS kits.

#### 10x Chromium library

Genomic DNA quality was assessed by pulsed-field gel electrophoresis to determine suitability for 10x Chromium library preparation (10x Genomics). A total of 1.125 ng of input was used for library preparation according to the manufacturer's instructions without size-selection. Final library concentration was determined by qPCR (KAPA Library Quantification Kit, Roche) and size-adjusted according to Bioanalyzer DNA 100 chip (Agilent) results. Next, 2 × 150 bp of Illumina PE sequencing with an 8-base index read was performed on the HiSeq 4000 using the HiSeq 3000/4000 Cluster and SBS kits.

#### Genome assembly

Genome assembly was completed using the DeNovoMAGIC software platform (NRGene). This is a proprietary DeBruijn-graph-based assembler that was used to produce assemblies of several challenging plant genomes such as corn [1] and ancestral wheat *Aegilops tauschii* [3]. The following outlines design of the assembler and steps of the assembly process.

#### Reads pre-processing

In the pre-processing step, we first removed PCR duplicate reads and trimmed Illumina adaptor AGATCGGAAGAGC and Nextera linker (for MP library) sequences. We then merged the PE 450 bp 2 × 250 bp overlapping reads with minimal required overlap of 10 bp to create stitched reads using the approach similar to the one implemented in the Flash software [28].

#### Error correction

We scanned through all merged reads to detect and filter out reads with apparent sequencing errors by examining *k*-mers (*k* = 24) in the reads and looking for low abundance *k*-mers. We have high coverage data (∼450x), with each read yielding 127 (150–24+1) to 227 (250–24+1) *k*-mers. Thus, average 24-mer coverage is at least 300x. The 24-mers that only appear fewer than 10 times in the set of reads likely contain errors. We did not use the reads that contain these low abundance *k*-mers for building initial contigs.

#### Contig assembly

The first step of the assembly consists of building a DeBruijn graph (*k*-mer = 127 bp) of contigs from all filtered reads. Next, PE and MP reads are used to find reliable paths in the graph between contigs for repeat resolving and contig extension.The 10x barcoded reads were mapped to contigs to ensure that adjacent contigs were connected only when there is evidence that those contigs originate from a single stretch of genomic sequence (reads from the same two or more barcodes were mapped to the same contigs).

#### Split phased/un-phased assembly processes

Two parallel assemblies take place to complete the phased and un-phased assembly result. The phased assembly process utilizes the complete set of contigs. In the un-phased assembly process, the homologous contigs are identified and one of the homologs is filtered out, leaving a subset of the homozygous and one of the homologous contigs in heterozygous regions. The linking information of both homologous contigs is kept through the assembly process of the un-phased assembly, usually enabling longer un-phased scaffolds.

#### Scaffolding

All of the following steps are done in parallel for both the phased and un-phased assemblies. Contigs were linked into scaffolds with PE and MP information, estimating gaps between the contigs according to the distance of PE and MP links. In addition, for the phased assembly, 10x data were used to validate and support correct phasing during scaffolding.

#### Gap filling

A final gap fill step used PE and MP links and DeBruijn graph information to locally construct a unique path through the graph connecting the gap edges. The path was used to close the gap if it was unique and its length was consistent with the gap size estimate.

#### Scaffold split/merge

We used 10x barcoded reads to refine and merge scaffolds. All barcoded 10x reads were mapped to the assembled scaffolds. Clusters of reads with the same barcode mapped to adjacent contigs in the scaffolds were identified to be part of a single long molecule. Next, each scaffold was scanned with a 20 kb length window to ensure that the number of distinct clusters that cover the entire window (indicating a support for this 20 kb connection by several long molecules) is statistically significant with respect to the number of clusters that span the left and the right edge of the window. If there was a statistically significant disagreement in the coverage by the clusters over the window, we broke the scaffold at the two edges of the window. Finally, the barcodes that were mapped to the scaffold edges (first and last 20 kb sequences) were compared to generate a graph of scaffolds. The scaffolds are nodes and the edges are links connecting nodes with more than two common barcodes on the ends. We broke the links to the nodes that had more than two links and output the resulting linear paths in the scaffold graph as final scaffolds.

## Summary

We show that Tur_tru_Illumina_hap_v1 is more complete and more accurate compared to the current best reference Tur_tru v1, based on the amount and composition of sequence, the consistency of the MP alignments to the assembled scaffolds, and on the analysis of conserved single-copy mammalian orthologs. The additional 12.5% of sequence data identified and assembled here was found to contain 165 additional BUSCO alignments as compared to the latest published assembly Tur_tru v1. The large scaffolds represented by Tur_tru_Illumina_hap_v1 enabled and confirmed expected synteny to human chromosome 1. The phased *de novo* assembly Tur_tru_Illumina_phased_v1 is of the first publicly available, and it provides the community with novel ways to explore the heterozygous nature of the dolphin genome. These findings illustrate the impact of improved sample preparation and improved *de novo* assembly methods on progress toward more complete and accurate reference quality genomes. Better-quality assemblies will improve our understanding of gene structure, function, and evolution in mammalian species.

## Availability of supporting data

The dolphin assembly Tur_tru_Illumina_hap_v1 has been deposited at NCBI under BioProject PRJNA476133, accession QMGA00000000. The dolphin assembly Tur_tru_Illumina_phased_v1 has been deposited at NCBI under BioProject PRJNA478376, accession QUXD00000000. All data are also available from the *GigaScience* GigaDB repository [29].

## Abbreviations

BUSCO: Benchmarking Universal Single-Copy Orthologs; MP: mate pair; NCBI: National Center for Biotechnology Information; Ns: ambiguous nucleotides; PE: paired end; SNP: single-nucleotide polymorphism.

## Competing interest

K.V. and C.T.L. were both full-time employees of Illumina at the time this work was completed. G.B.Z., K.B., and T.B. are employees of NRGene, a company that provides software analysis tools for *de novo* assembly.

## Author contributions

K.V.M., C.T.L., and M.M.V. designed the project. K.V.M., C.T.L., and A.Z. wrote the manuscript. G.B.Z., T.B., and K.B. generated genome assemblies. A.Z. conducted validation, MP consistency analysis, human chromosome 1 and BUSCO analyses, and submitted the genomes to NCBI. J.S.L. provided the blood sample. J.L., A.N., M.R., M.G., E.J., and B.S. processed samples, generated sequencing, and completed quality checks on sequence data. All authors contributed to editing the manuscript.

## Supplementary Material

GIGA-D-18-00268_Original_Submission.pdf

GIGA-D-18-00268_Revision_1.pdf

Response_to_Reviewer_Comments_Original_Submission.pdf

Reviewer_1_Report_Original_Submission -- Granger Gideon Sutton, Ph.D. 8/17/2018 Reviewed

Reviewer_2_Report_Original_Submission -- Shengfeng Huang 8/24/2018 Reviewed

## References

[1] Hirsch CN, Hirsch CD, Brohammer AB, et al. Draft assembly of elite inbred line PH207 provides insights into genomic and transcriptome diversity in maize. Plant Cell. 2016;28(11):2700–14. 27803309 10.1105/tpc.16.00353 PMC5155341

[2] Avni R, Nave M, Barad O, et al. Wild emmer genome architecture and diversity elucidate wheat evolution and domestication. Science. 2017;357(6346):93–97. 28684525 10.1126/science.aan0032

[3] Luo MC, Gu YQ, Puiu D et al. Genome sequence of the progenitor of the wheat D genome *Aegilops tauschii*. Nature. 2017;551(7681):498–502. 29143815 10.1038/nature24486 PMC7416625

[4] Zimin A, Stevens KA, Crepeau MW et al. An improved assembly of the loblolly pine mega-genome using long-read single-molecule sequencing. GigaScience. 2017;6(1):1–4. 10.1093/gigascience/giw016 PMC5437942 28369353

[5] Genome 10K Community of Scientists. Genome 10K: a proposal to obtain whole-genome sequence for 10000 vertebrate species. J Hered. 2009;100(6):659–74. 19892720 10.1093/jhered/esp086 PMC2877544

[6] Koepfli K, Paten B, Antunes A, et al. The Genome 10K project: a way forward further. Annual Review of Animal Biosciences. 2015;3:57–111. 25689317 10.1146/annurev-animal-090414-014900 PMC5837290

[7] Lewin HA, Robinson GE, Kress WJ et al. Earth BioGenome project: sequencing life for the future of life. Proc Natl Acad Sci. 2018;115(17):4325–33. 29686065 10.1073/pnas.1720115115 PMC5924910

[8] Mohr DW, Naguib A, Weisenfeld N, et al. Improved *de novo* genome assembly: Linked-Read sequencing combined with optical mapping produce a high quality mammalian genome at relatively low cost. bioRxiv. 2017:128348 10.1101/128348.

[9] Armstrong EE, Taylor RW, Prost S et al. Cost-effective assembly of the African wild dog (*Lycaon pictus*) genome using linked reads. GigaScience. 2018 10.1093/gigascience/giy124. PMC6350039 30346553

[10] Hammond PS, Bearzi G, Bjørge A, et al. Tursiops truncatus. The IUCN Red List of Threatened Species 2012:e.T22563A17347397. 10.2305/IUCN.UK.2012.RLTS.T22563A17347397.en, Accessed 15 June 2018.

[11] Rosel PE, Hancock‐Hanser BL, Archer FI, et al. Examining metrics and magnitudes of molecular genetic differentiation used to delimit cetacean subspecies based on mitochondrial DNA control region sequence. Special Issue: Delimiting subspecies using primarily genetic data. Marine Mammal Science. 2017;33(S1):76–100.

[12] McGowen MR, Grossman LI, Wildman DE Dolphin genome provides evidence for adaptive evolution of nervous system genes and a molecular rate slowdown. Proc R Soc B. 2012;279(1743):3643–51. 10.1098/rspb.2012.0869 PMC3415902 22740643

[13] Venn-Watson S, Carlin K, Ridgway S Dolphins as animal models for type 2 diabetes: sustained, post-prandial hyperglycemia and hyperinsulinemia. Gen Comp Endocrinol. 2011;170(1):193–9. 20951701 10.1016/j.ygcen.2010.10.005

[14] Venn-Watson S, Smith CR, Stevenson S et al. Blood-based indicators of insulin resistance and metabolic syndrome in bottlenose dolphins (*Tursiops**truncatus*). Front Endocrinol (Lausanne). 2013;4:136. 24130551 10.3389/fendo.2013.00136 PMC3793200

[15] Venn-Watson S Dolphins and diabetes: Applying one health for breakthrough discoveries. Frontiers in Endocrinology. 2014;5(227). 10.3389/fendo.2014.00227. PMC4273662 25566195

[16] Neely BA, Debra L, Ellisor DL et al. Proteomics as a metrological tool to evaluate genome annotation accuracy following de novo genome assembly: a case study using the Atlantic bottlenose dolphin (*Tursiops**truncatus*). bioRxiv. 2018:254250 10.1101/254250. PMC10531373 37761836

[17] Sobolesky P, Parry C, Boxall B et al. Proteomic analysis of non-depleted serum proteins from bottlenose dolphins uncovers a high vanin-1 phenotype. Sci Rep. 2016;26(6):33879. 10.1038/srep33879 PMC5036180 27667588

[18] Venn-Watson S, Smith CR, Gomez F, et al. Physiology of aging among healthy, older bottlenose dolphins (*Tursiops**truncatus*): comparisons with aging humans. J Comp Physiol B. 2011;181(5):667–80. 21253749 10.1007/s00360-011-0549-3

[19] Lindblad-Toh K, Garber M, Zuk O, et al. A high-resolution map of human evolutionary constraint using 29 mammals. Nature. 2011;478(7370):10530. 10.1038/nature10530 PMC3207357 21993624

[20] Foote AD, Liu Y, Thomas GWC et al. Convergent evolution of the genomes of marine mammals. Nat Genet. 2014;47(3):272–5. 10.1038/ng.3198 PMC4644735 25621460

[21] Marks P, Garcia S, Alvaro Martinez A et al. Resolving the full spectrum of human genome variation using linked-reads. bioRxiv. 2018:230946 10.1101/230946 PMC6442396 30894395

[22] Bielec PE, Gallagher DS, Womack JE, et al. Homologies between human and dolphin chromosomes detected by heterologous chromosome painting. Cytogenet Genome Res. 1998;81(1):18–25. 10.1159/000015002 9691170

[23] Murphy WJ, Frönicke L, O'Brien SJ, et al. The origin of human chromosome 1 and its homologs in placental mammals. Genome Res. 2003;13(8):1880–8. 12869576 10.1101/gr.1022303 PMC403779

[24] Marçais G, Delcher AL, Phillippy AM, et al. MUMmer4: a fast and versatile genome alignment system. PLOS. 2018;14(1):1005944. 10.1371/journal.pcbi.1005944 PMC5802927 29373581

[25] Langmead B, Salzberg SL Fast gapped-read alignment with Bowtie 2. Nat Methods. 2012;9(4):357–9. 22388286 10.1038/nmeth.1923 PMC3322381

[26] Putnam NH, O'Connell BL, Stites JC et al. Chromosome-scale shotgun assembly using an in vitro method for long-range linkage. Genome Res. 2016;26(3):342–50. 26848124 10.1101/gr.193474.115 PMC4772016

[27] Schneider VA, Graves-Lindsay T, Howe K, et al. Evaluation of GRCh38 and de novo haploid genome assemblies demonstrates the enduring quality of the reference assembly. Genome Res. 2017;27(5):849–64. 28396521 10.1101/gr.213611.116 PMC5411779

[28] Magoč T, Salzberg SL FLASH: fast length adjustment of short reads to improve genome assemblies. Bioinformatics. 2011;27(21):2957–63. 21903629 10.1093/bioinformatics/btr507 PMC3198573

[29] Viaud-Martinez KA, Lawley CT, Martinez Vergara M, et al. Supporting data for “New de novo assembly of the Atlantic bottlenose dolphin (*Tursiops truncatus*) improves genome completeness and provides haplotype phasing.” GigaScience Database, 2018 10.5524/100546

