# Supplementary material for: New de novo assembly of the Atlantic bottlenose dolphin (Tursiops truncatus) improves genome completeness and provides haplotype phasing
Source: Gigascience. 2019 Jan 29;8(3):giy168. doi: 10.1093/gigascience/giy168 (PMC6443575; doi:10.1093/gigascience/giy168)
Supplement: GIGA-D-18-00268_Revision_1.pdf [file giy168_giga-d-18-00268_revision_1.pdf]

GigaScience

New de novo assembly of the Atlantic bottlenose dolphin (Tursiops truncatus) improves genome completeness and provides haplotype phasing

--Manuscript Draft--

**Manuscript Number:**	GIGA-D-18-00268R1
**Full Title:**	New de novo assembly of the Atlantic bottlenose dolphin (Tursiops truncatus) improves genome completeness and provides haplotype phasing
**Article Type:**	Data Note
**Funding Information:**	USDA (2018-67015-28199) — Dr Aleksey V Zimin
**Abstract:**	High quality genomes are essential to resolve challenges in breeding, comparative biology, medicine and conservation planning. New library preparation techniques along with better assembly algorithms result in continued improvements in assemblies for non-model organisms, moving them toward reference quality genomes. We report on the latest genome assembly of the Atlantic bottlenose dolphin leveraging Illumina sequencing data coupled with a combination of several library preparation techniques. These include Linked-Reads (Chromium, 10x Genomics), mate pairs, long insert paired ends and standard paired ends. Data were assembled with the commercial DeNovoMAGICTM assembly software resulting in two assemblies, a traditional "haploid" assembly (Tur_tru_Illumina_hap_v1) that is a mosaic of the two parental haplotypes and a phased assembly (Tur_tru_Illumina_phased_v1) where each scaffold has sequence from a single homologous chromosome. We show that Tur_tru_Illumina_hap_v1 is more complete and accurate compared to the current best reference based on the amount and composition of sequence, the consistency of the mate pair alignments to the assembled scaffolds, and on the analysis of conserved single-copy mammalian orthologs. The phased de novo assembly Tur_tru_Illumina_phased_v1 is the first publicly available for this species and provides the community with novel and accurate ways to explore the heterozygous nature of the dolphin genome.
**Corresponding Author:**	Aleksey Zimin<br><br>UNITED STATES
**Corresponding Author Secondary Information:**	
**Corresponding Author's Institution:**	
**Corresponding Author's Secondary Institution:**	
**First Author:**	Karine A. Martinez-Viaud
**First Author Secondary Information:**	
**Order of Authors:**	Karine A. Martinez-Viaud
	Cindy Taylor Lawley
	Milmer Martinez Vergara
	Gil Ben-Zvi
	Tammy Biniashvili
	Kobi Baruch
	Judy St. Leger
	Jennie Le
	Aparna Natarajan
	Marlem Rivera

Powered by Editorial Manager® and ProduXion Manager® from Aries Systems Corporation

	Marbie Guillergan
	Erich Jaeger
	Brian Steffy
	Aleksey V Zimin
**Order of Authors Secondary Information:**	
**Response to Reviewers:**	We also included a Word copy of this document in the re-submission.

We thank the reviewers for their careful review of the paper and constructive comments. We feel that addressing the comments has made the manuscript significantly stronger. We updated the tables and added two additional figures to the manuscript. Below we provide point-by-point responses to the reviewers' reports and list changes to the text that we have made in response to the comments. We have made changes to the paper using the "Track changes" feature in Word and we submit the revised version with changes highlighted. We also listed major changes in the responses.

Reviewer #1: "New de novo assembly of the Atlantic bottlenose dolphin (Tursiops truncatus) improves genome completeness and provides haplotype phasing" reports on improved haploid and diploid assemblies for the dolphin. The two major claims are: "Tur_tru_Illumina_hap_v1 is more complete and accurate compared to the current best reference based on the amount and composition of sequence, the consistency of the mate pair alignments to the assembled scaffolds, and on the analysis of conserved single-copy mammalian orthologs", and "Tur_tru_Illumina_dip_v1 is of the highest quality available for this species and provides the community with novel and accurate ways to explore the heterozygous nature of the dolphin genome".
This paper does not compare different sequencing technologies for sequencing the same genome such as 10X versus Oxford Nanopore. Nor does it compare different assemblers using the same data. This is a simple straight forward paper reporting an improved haplotype assembly and the first partially resolved diploid assembly for T. truncates. The three key sections are: Genome assembly comparison, Assembly validation through MP consistency, and Haplotype resolution. In the Genome assembly comparison section, the best existing assembly is aligned to the haplotype assembly using the MUMmer4 package which shows that there is much more unique sequence in the new assembly while maintaining similar contiguity statistics. BUSCO analysis shows that this unique additional sequence includes valuable protein coding gene regions. The new assembly is not missing any BUSCO genes from the existing assembly. The conclusion that the haplotype assembly is an improvement seems valid although the
authors note there is some failure to collapse haplotypes 34 additional BUSCO genes are duplicated in the new "haplotype" assembly. Some BUSCO analysis of the diploid genome is also presented which is not as encouraging with 393 missing BUSCOs and only 2079 of the 3371 found BUSCOs duplicated showing that only partial haplotype resolution is achieved. In the Assembly validation through MP consistency section, the MP analysis shows that the new assembly is better than the existing assembly and plausible reasons for that are hypothesized.
1.The authors should however include a caveat that since the species level structural and transposon insertional variation is not known and since different individuals were assembled that some of the better MP statistics for the new assembly could be due to the use of read pairs from the individual sequenced for the new assembly.

Response:
Starting Line 214, We revised the text to: … Tur_tru v1 compared to Tur_tru_Illumina_hap_v1 (Table 4). Of course, some level of discrepancy is expected, because the two assemblies represent two different individuals with unknown level of structural variation between them. However, in concert, the two different categories may also suggest a possibility of a relatively higher number of locally mis-ordered or misoriented contigs in the scaffolds of Tur_tru v1 assembly. …

2.A major problem however is that table 4 does not support the text or at least is not explained in the text. Some
category of mate pair status must be being left out since there are many more mate

pairs claimed in various categories for Tur_tru_v1 than for Tur_tru_Illumina_hap_v1. Based on the text one would expect more "Same scaffold happy" MPs for Tur_tru_Illumina_hap_v1 but this is not the case. Also the text claims Tur_tru_v1 has 8 times more "Same scaffold misoriented" MPs when it doesn't even have 7 times as many.

Response:
We agree with the reviewer that there are more mate pairs claimed in all categories for Tur_tru_v1. The difference can be explained by the peculiarities of the MUMmer software that we used to produce the alignments. By default, if there is a high-identity repeat region in the assembly, Nucmer will not find any seeds for the alignment, because all alignments are initiated by looking for clusters of exact matching seeds in the reference genome (genome we align to) with minimum length of 20. If the reference has two regions of length >=20 that are identical, no seeds will be found there. Thus, an assembly that has two or more copies of repeat will have no reads mapping to either copy, while the assembly that collapsed repeat to one copy will have reads mapping to the same copy. To validate this hypothesis, we re-did the alignments using Bowtie2 short read aligner to align the same data to both assemblies. Bowtie2 does not have the same deficiency as MUMmer in mapping reads to repeat regions. We only used alignments of reads that mapped uniquely to the assembly to avoid noise in the results. All numbers in Table 4 have been updated, and we added new category where we list the sum of all mate pairs aligned to the same scaffold. Now we see that the number of mates aligned to both assemblies is similar, with alignment patterns that support the reasoning in the text. We updated the text to replace MUMmer4 by Bowtie2. We also added the analysis of the mate pair alignments to the haplotype phased assembly.

Starting Line 232 We revised the text to: The haplotype phased assembly is much more fragmented, resulting in a higher relative number of mate pairs mapping to different scaffolds. However, when looking at the “internal” mate pairs, i.e. where both mates map at least 10Kb away from the scaffold ends, we see remarkable consistency with less than 0.5% of the mates mapped to the wrong scaffold (see next section). Since for this analysis we only used mates mapping uniquely to the assembly, and there are two copies of the genome in the assembly, the total number of mapped mates is much lower.

3.Finally in the Haplotype resolution system, MPs were again used to evaluate the amount of haplotype switching between scaffolds asserted to be haplotype separated. No MPs were found to map to different haplotype separated scaffolds. The problem with this section is that there may be ascertainment bias in that only large separated scaffolds were used. The number of MPs mapped is very small compared to the numbers in table 4 and again there is no accounting given for what happened to most mate pairs which should be covered in a table 5. I would assume that smaller scaffolds are much more likely to not be separated as well. The same mate pair library was mapped to the entire haplotype assembly so why not the entire diploid assembly? Also there is no evaluation of comparing the diploid assembly to the haploid assembly. Large separated scaffolds were apparently mapped but not smaller ones?
Response:
In haplotype separation it is typically easier to phase short regions (an example is a single SNP that is different between the two haplotypes, or in insertion in one of the haplotypes). However, when phasing large regions one has to be careful not to “jump” to a different haplotype while extending haploid sequence. This is why we originally looked at the largest haplotype-phased scaffolds for evaluation of the efficacy of haplotype separation. We changed the way we measure the efficacy of the haplotype resolution; the new text and results are as follows.
Starting Line 273 We changed the text to: We analyzed the quality of the haplotype phasing as follows. In haplotype phasing it is easy to phase small regions. For example, a single isolated SNP with no haplotype differences within 100 bp in both directions, can be trivially phased into two 201bp (or longer) contigs different by one base in the middle. It gets more difficult for larger contigs/scaffolds, where one must make sure that the contig/scaffold represents single haplotype and not a “mosaic” of haplotypes, that is the SNPs and other bigger haplotype differences are correctly “phased”. To do that we mapped the mate pairs from the 5-7Kb mate pair library to all phased scaffolds using Bowtie2 [28], and then examined the “internal” mate pairs

Powered by Editorial Manager® and ProduXion Manager® from Aries Systems Corporation

where both reads in each pair mapped to the assembly, and one read mapped within 10Kb away from the ends of the scaffold. This would imply that the other mate must map to the same scaffold and not its haplotype, if haplotype phasing is done properly. If it does not, then it indicates an apparent mis-assembly or failure to phase haplotypes. By measuring the number of “properly” aligned internal mates, where both mates aligned to the same scaffold vs. “improper” internal mates where the mates aligned to different scaffolds, one can measure the efficacy of the haplotype phasing. There were 35,697,369 pairs where both mates mapped properly to the same scaffold, while only 169,244 mapped improperly, that is to two different scaffolds. The percentage of improperly mapping mate pairs is only 0.5%, indicating that haplotype resolution was done properly.

4.In table 2 it is not clear if total sequence for the scaffolds includes Ns in the gaps but it would appear to. There is no discussion of why the diploid assembly is more than twice as big as the haploid assembly given that based on BUSCO the haplotype resolution is far from complete. Assuming gaps are included in the sizes the explanation seems obvious that many scaffolds overlap with or are contained in other scaffolds with contigs interleaving.
I cannot recommend accepting this article until the discrepancies in table 4 are explained and a comparison of the haploid and diploid assemblies is given to try to explain the amount of haplotype resolution and the scaffold size discrepancy.

Response:
The statistics for the phased assembly in Table 2 were computed incorrectly. Thank you for helping us identify this discrepancy. They were computed on the pre-release draft version of the assembly that had redundant sequences. We re-computed the statistics on the latest version of the assembly that was generated after the initial writing of that section of the paper and updated Table 2. The latest version is available from our ftp site listed in the paper and has been resubmitted to NCBI with accession QUXD00000000. We clarified the caption in Table 2 by adding “The total sequence listed excludes Ns.” For confirmation, we re-computed the numbers to make sure N’s are excluded from all N50 and size computations, because N50 numbers should reflect sizes of actual sequences without (sometimes) arbitrarily estimated gaps. Also we updated the numbers for the haploid assembly since they changed slightly after filtering the assembly for contaminants, which is part of NCBI submission process. The filtering results were reported to us after we submitted the manuscript.

5.I also strongly recommend that the authors tone down any claims for superiority of this assembly since it is not that much better than the existing one - better but not perfect. For example, the diploid assembly is touted as the "highest quality available for this species" but then later in the paper it is claimed it is the only diploid assembly for the species which makes the previous claim trivially true but misleading.

Response:
We agree that our text sounded misleading and we thank the reviewer for bringing that to our attention. Scientific writing should be state the facts clearly. We have changed the “highest” to the “first” in the following sentence in the abstract, and in the summary, and it now reads:

Starting Line 47 We changed the text to: The phased de novo assembly Tur_tru_Illumina_dip_v1 is the first publicly available for this species and provides the community with novel and accurate ways to explore the heterozygous nature of the dolphin genome.

Reviewer #2: This manuscript provide new genome assemblies for Atlantic bottlenose dolphin based on new sequencing data set. Here are my comments:

1) It appears that the new assemblies did not leverage the old but valuable sequencing data based on Sanger and 454 reads, which together give about ~6X coverage of data. Such data might benefit the contiguity of the assembly a lot. And this is probably already reflected on the contig N50 size, which is only 30Kb in the new assembly (even hundreds coverage of reads were used), shorter than the previous assemblies based on the 454/sanger reads (37Kb). The authors need to discuss on this.

Powered by Editorial Manager® and ProduXion Manager® from Aries Systems Corporation

	Response:<br>The old 454 and Sanger data was from a different animal. Using it would likely fracture assembly even further because of the structural/haplotype differences between the animals. Typically for a good assembly data from only one individual must be used.<br><br>2) It is not very easy to get the detailed information on what software were used on scaffolding, gap filling, phasing and scaffold splitting/merging. Did the authors used a home-made software, or publicly available tools? Where to access these tools? what parameters were used?<br><br>Response:<br>The software that was used for this assembly is DeNovoMagic, it is proprietary and it was developed by NRGene LTD. In the paper we outline the steps that were followed by the software to produce the assembly. The software is not open source and it is not available for general use. However, it has been used before to produce many genome assemblies, such as maize and wheat, published now in major journals.<br><br>3) The names of Tur_tru_Illumina_hap_v1 and Tur_tru_Illumina_dip_v1 could be somewhat misleading: hap=haploid whereas the dip=diploid. May be haploid versus phased-haplotypes.<br><br>Response:<br>We understand the confusion. We changed the name of the phased assembly to Tur_tru_Illumina_phased_v1<br><br>4) The synteny analysis appears show no new finding or improvement over the old assembly (which may be due to the fact the old ones are already chromosome-level assembly?). If there is new findings/improvements, the authors are encouraged to show them.<br><br>Response:<br>Neither the old nor the new assembly are chromosome-level. Both are scaffold level assemblies. We show the synteny to human to illustrate the finding mentioned in the earlier literature.<br><br>5) There could be more comparative analysis between the new and old assemblies. It appears they are based on different data set. For example, some Venn figures could to use to show the differences between them. LIke, 1000 genes, 900 are both complete in new and old assemblies, while new assemblies has 60 more complete genes and the old have 40 more complete genes but they are not overlapped. And some examples of the genes could be provided.<br><br>Response:<br>This is an excellent suggestion, and we thank the reviewer for bringing this clarifying idea to our attention. We added the Venn diagram with the analysis of BUSCO genes to the paper as Figure 1.<br><br>--
**Additional Information:**	
**Question**	**Response**
Are you submitting this manuscript to a special series or article collection?	No
**Experimental design and statistics**<br><br><br>Full details of the experimental design and statistical methods used should be given in the Methods section, as detailed in our Minimum Standards Reporting Checklist.	Yes

Information essential to interpreting the data presented should be made available in the figure legends.<br><br>Have you included all the information requested in your manuscript?	
**Resources**<br><br>A description of all resources used, including antibodies, cell lines, animals and software tools, with enough information to allow them to be uniquely identified, should be included in the Methods section. Authors are strongly encouraged to cite Research Resource Identifiers (RRIDs) for antibodies, model organisms and tools, where possible.<br><br>Have you included the information requested as detailed in our Minimum Standards Reporting Checklist?	Yes
**Availability of data and materials**<br><br>All datasets and code on which the conclusions of the paper rely must be either included in your submission or deposited in publicly available repositories (where available and ethically appropriate), referencing such data using a unique identifier in the references and in the "Availability of Data and Materials" section of your manuscript.<br><br>Have you have met the above requirement as detailed in our Minimum Standards Reporting Checklist?	Yes

Manuscript
Click here to access/download;Manuscript;GIGAScience_v26_final-
Click here to view linked References

New de novo assembly of the Atlantic bottlenose dolphin (*Tursiops truncatus*) improves genome completeness and provides haplotype phasing.

Karine A. Martinez-Viaud *[1], Cindy Taylor Lawley*[2,6], Milmer Martinez Vergara*[3], Gil Ben-Zvi[4], Tammy Biniashvili[4], Kobi Baruch[4], Judy St. Leger[5], Jennie Le[1], Aparna Natarajan [1], Marlem Rivera[1,6], Marbie Guillergan[1], Erich Jaeger [1], Brian Steffy [1] and Aleksey Zimin[7]

1. Illumina, Inc, San Diego, CA 92122, USA
2. GinkgoFish LLC, Carson City, NV 89703
3. Plant with Purpose, San Diego, CA 92117, USA
4. NRGene, Ness-Ziona, 7403649, Israel
5. SeaWorld San Diego, San Diego, CA 92109, USA
6. Ocean Discovery Institute, San Diego, CA 92109 USA
7. Johns Hopkins University, Baltimore, MD 21205, USA

*Authors contributed equally to the project
**Corresponding authors: kviaud@illumina.com and alekseyz@jhu.edu

Karine A. Martinez-Viaud kviaud@illumina.com
Cindy Taylor Lawley cindylawleyphd@gmail.com
Milmer Martinez Vergara milmer@plantwithpurpose.org
Gil Ben-Zvi gil@nrgene.com, ORCID: 0000-0002-8649-6531
Tammy Biniashvili tammy@nrgene.com, ORCID: 0000-0003-3053-2799
Kobi Baruch kobi@nrgene.com, ORCID: 0000-0002-0193-9621
Judy St. Leger Judy.St.Leger@SeaWorld.com, ORCID: 0000-0002-6213-3429
Jennie Le jle@illumina.com
Aparna Natarajan anatarajan1@illumina.com
Marlem Rivera mrivera@illumina.com
Marbie Guillergan mguillergan@illumina.com
Erich Jaeger ejaeger@illumina.com
Brian Steffy bsteffy@illumina.com
Aleksey Zimin alekseyz@jhu.edu, ORCID: 0000-0001-5091-3092

Abstract

High quality genomes are essential to resolve challenges in breeding, comparative biology, medicine and conservation planning. New library preparation techniques along with better assembly algorithms result in continued improvements in assemblies for non-model organisms, moving them toward reference quality genomes. We report on the latest genome assembly of the Atlantic bottlenose dolphin leveraging Illumina sequencing data coupled with a combination of several library preparation techniques. These include Linked-Reads (Chromium, 10x Genomics), mate pairs, long insert paired ends and standard paired ends. Data were assembled with the commercial DeNovoMAGIC™ assembly software resulting in two assemblies, a traditional "haploid" assembly (Tur_tru_Illumina_hap_v1) that is a mosaic of the two parental haplotypes and a phased assembly (Tur_tru_Illumina_phased_v1) where each scaffold has sequence from a single homologous chromosome. We show that Tur_tru_Illumina_hap_v1 is more complete and accurate compared to the current best reference based on the amount and composition of sequence, the consistency of the mate pair alignments to the assembled scaffolds, and on the analysis of conserved single-copy mammalian orthologs. The phased de novo assembly Tur_tru_Illumina_phased_v1 is the first publicly available for this species and provides the community with novel and accurate ways to explore the heterozygous nature of the dolphin genome.

Keywords: de novo genome assembly, bottlenose dolphin, Tursiops truncatus, 10x Genomics, DeNovoMAGIC™, Illumina

Introduction

Technical advances in the past decade have reduced sequencing costs and improved access to sequencing data. Subsequent improvements in DNA extraction, preparation, and assembly algorithms facilitate low cost accurate de novo genome assemblies. Such assemblies are essential for constructing haplotype diversity databases for breeding, comparative biology, medicine and conservation planning. Even highly complex genomes now benefit from higher contiguity and improved protein coding coverage [1-4]. Consortium efforts to catalogue biodiversity of pivotal species of comparative evolutionary significance will continue to drive novel low-cost approaches toward reference quality assemblies with chromosome level resolution [5-7]. Here we use a combination of methods to drive improvements in assembly structure for the Atlantic bottlenose dolphin (*Tursiops truncatus*, NCBI:txid9739). This genome assembly, like that of the Hawaiian Monk seal and African wild dog, is being published with the goal to facilitate research on comparative genomics, provide structure for cataloging biodiversity and ultimately support decisions around species conservation and management [8, 9].

The bottlenose dolphin is one of the most widely studied marine mammals, however the taxonomy of the *Tursiops* genus remains unresolved. Numerous species designations have been suggested but not adopted due to a lack of resolution afforded by available data [10]. Even with new molecular genetic markers, we have reached a limitation on resolution from genetic data available to delineate species, subspecies and populations [11]. To usher this species into the era of genomics, a high-quality reference genome is essential. It provides structure to catalogue diversity within and between species at the whole genome level. In addition, the parallel molecular trajectory between dolphin and other mammalian species [12] makes the bottlenose dolphin a useful model to understand aspects of human health such as metabolic processes/diabetes [13-15], proteomics [16, 17] and aging [18].

A preliminary dolphin genome was first submitted to NCBI (TurTru1.0; GCA_000151865.1) using low coverage (2.82X) Sanger sequencing for the purpose of cross-species comparison [12, 19, 20]. Subsequent improvements were achieved through the addition of 30X Illumina short read data and 3.5X 454 data (Ttru_1.4; GCA_000151865.3). A much more complete genome was submitted in 2016 leveraging improvements in library preparation and assembly methods (Meraculous v. 2.2.2.5 and HiRise v. 1.3.0-116-gf50c3ce; Dovetail, Inc) with 114X coverage of Illumina HiSeq data prepared with proximity ligation Hi-C protocol (Tur_tru v1; GCA_001922835.1; [16]).

With the collection of data from multiple sources including Linked-Reads (Chromium, 10x Genomics; [21]), mate pairs (MP), long insert paired ends and standard paired ends, and the DeNovoMAGIC assembly tool (NRGene, Ness-Ziona, Israel), we provide an improved haploid reference quality dolphin genome assembly as well as the first haplotype phased diploid assembly. We refer to our unphased assembly as Tur_tru_Illumina_hap_v1 and to the phased assembly as Tur_tru_Illumina_phased_v1. Using Tur_tru v1 for comparison, our assembly shows increased contiguity and completeness with high consistency to the MP data and orthologous mammalian protein alignments. Additionally, by aligning Tur_tru_Illumina_hap_v1 to the Human reference genome, we illustrate the synteny of the dolphin scaffolds to human chromosome 1 [22, 23].

Results

Coverage. We generated sequence data for a total coverage of approximately 450X, the majority from PCR Free and Chromium 10X Genomics Linked-Read libraries (Table 1). Coverage was computed using 2.4Gbp estimated genome size. Genome assembly was conducted using DeNovoMAGIC^{TM} software (NRGene, Ness-Ziona, Israel). More detail about the library preparation and the assembly process are found in the Methods section.

Haploid and diploid assemblies. We report on two assemblies in this manuscript, one traditional haploid consensus assembly Tur_tru_Illumina_hap_v1 that represents a mosaic of the maternal and paternal haplotypes, and the other haplotype-phased (i.e., diploid) assembly where each scaffold represents sequence corresponding to a single haplotype, Tur_tru_Illumina_phased_v1. The quantitative statistics for both assemblies are listed in Table 2. The phased or diploid genome assembly was made possible using Illumina sequencing data by leveraging the combination of library prep methods including Linked-Reads, is a significant advance and will provide the community with a powerful genomic tool for the downstream analysis in the context of the true heterozygous dolphin genome.

Genome assembly comparison. Both assemblies were compared to the best available assembly Tur_tru v1 (NCBI accession GCA_001922835.1; [16]). We did not use the Ttru_1.4 assembly (NCBI accession GCA_000151865.3) because the contiguity statistics of the Ttru_1.4 are vastly inferior to the Tur_tru v1 with a contig N50 3 times smaller than Tur_tru v1 and scaffold N50 over 200 times smaller.

The statistics for the Tur_tru_Illumina_hap_v1 assembly show bigger scaffolds but slightly smaller contigs with about 13% more sequence in the scaffolds compared to Tur_tru v1 (Table 2). More sequence does not necessarily make for a better assembly considering that the extra sequence may be duplicated haplotypes or contaminants that do not belong to the original organism. To characterize the extra sequence, we first aligned the Tur_tru_Illumina_hap_v1 to the Tur_tru v1 assembly using the Nucmer aligner which is part of MUMmer4 package [24]. We used default settings for generating the alignments. We then analyzed the alignments using the dnadiff package included with MUMmer4. 87.5% of Tur_tru_Illumina_hap_v1 sequence aligned to 97.9% of Tur_tru v1. This shows that 12.5% of Tur_tru_Illumina_hap_v1 had no alignments to Tur_tru v1, while only 2.1% of Tur_tru v1 had no alignments to Tur_tru_Illumina_hap_v1. Therefore, there are 301Mbp of extra novel sequence in our new assembly Tur_tru_Illumina_hap_v1. We then used the BUSCO (BUSCO, RRID:SCR_015008) tool to show that the extra sequence is meaningful (Table 3). Tur_tru_Illumina_hap_v1 had 160 missing BUSCOs, compared to 270 missing in Tur_tru v1. The number of duplicated BUSCOs was higher by only 34 in our assembly compared to Tur_tru v1. This suggests that most of the extra sequence in Tur_tru_Illumina_hap_v1 is not contamination or redundant sequence, and likely contains useful coding information. There were 105 BUSCOS missing from both assemblies. We examined the locations of the 165 BUSCOs that are only found in the Tur_tru_Illumina_hap_v1 and all of them fully or partially aligned to locations in the sequences that were missing in Tur_tru v1 assembly. Figure 1 shows the Venn diagram of BUSCOs aligned to both assemblies, showing that there are 165 BUSCOS that are only present in Tur_tru_Illumina_hap_v1 and 55 that are only present in Tur_tru v1, with 3779 present in both assemblies. The haplotype-resolved assembly is more fragmented and it is missing 266 BUSCOs. As expected most of the complete BUSCOs that were found (3537) are duplicated 2227), since they are found in different haplotypes.

Assembly validation through MP consistency. Since both Tur_tru v1 and Tur_tru_Illumina_hap_v1 reference the same species, we expect few rearrangements between the assemblies. To examine this, we compared the absolute and relative correctness of the scaffolds of Tur_tru_Illumina_hap_v1 assembly by aligning the Illumina data from the 5-7Kbp MP library to the scaffolds of

Tur_tru_Illumina_hap_v1, Tur_tru_Illumina_phased_v1,and Tur_tru v1 assemblies using the Bowtie2 (Bowtie 2, RRID:SCR_016368) tool [28]. We chose this library because it contained the largest number of valid 5-7Kbp mate pairs. We then used only high quality uniquely aligning mated reads (both mates had to align uniquely with quality score 42 in the SAM file) and classified the alignments of the MPs into the following categories (Table 4):

1. **Same scaffold happy** – number of MPs where both mates aligned to the same scaffold in the correct orientation with mate separation within 3 standard deviations of the library mean
2. **Same scaffold short** -- number of MPs where both mates aligned to the same scaffold in the opposite orientation with mate separation of less than 1000bp; these MPs are not indicative of scaffolding misassemblies, they are simply a byproduct of the mate pair library preparation process as they are MPs that are missing the circularization junction site between the mates
3. **Same scaffold long** – number of MPs where both mates aligned to the same scaffold in the correct orientation, but the mate separation exceeded three standard deviations of the library mean
4. **Same scaffold misoriented** -- number of MPs where both mates aligned to the same scaffold in the opposite orientation with mate separation of more than 1000bp
5. **Mates aligned to different scaffolds** – number of MPs where the two mates aligned to different scaffolds
6. **Only one mate in the pair aligned** – number of MPs where only one read aligned to the assembly.

“Same scaffold ALL” category in table 4 is the sum of all mates in categories 1 to 4, it is listed for completeness.

Comparing Tur_tru_Illumina_hap_v1 with Tur_tru v1, the total number of reads uniquely aligning to both “haploid” assemblies is very similar: about 295.2M reads aligned to Tur_tru_Illumina_hap_v1 vs. about 293.6M reads aligned to Tur_tru v1. The total number of mate pairs aligning to the same scaffold is larger for Tur_tru_Illumina_hap_v1. Of the mate pairs aligning to the same scaffold, the number of mate pairs in the ”Same scaffold happy” category is very similar between the two assemblies. The differences that stand out are the much larger (7.3 times more) number of mates that aligned to the same scaffold in the wrong orientation and the much larger (3.3 times more) number of the same scaffold long pairs in Tur_tru v1 compared to Tur_tru_Illumina_hap_v1 (Table 4). Of course, some level of discrepancy is expected, because the two assemblies represent two different individuals with unknown level of structural variation between them. However, in concert, the two different categories may also suggest a possibility of a relatively higher number of locally mis-ordered or misoriented contigs in the scaffolds of Tur_tru v1 assembly. This may be due to the scaffolding process used to create Tur_tru v1 assembly. The assembly was created with the HiRise assembler [25] using proximity ligation Hi-C data for scaffolding. The proximity ligation data provide MPs of all possible sizes, however, the MP distances and mate orientations are unknown. Since there are more shorter pairs than longer pairs due to the 3D structure of the DNA – it is much more likely to ligate parts of DNA that are closer to each other than the ones that are far apart. This property enables one to use these data for scaffolding. By mapping the pairs to the assembled scaffolds, one can measure how the distance between the mates in a pair varies with the number of pairs whose ends map to the same location in the assembly. However, the dependence is weak on the short end, meaning that the number of pairs of about 10Kbp in length is not much different from the number of links of 12-13Kbp in length. This frequently results in mis-orientations and shuffling of scaffold positions for contigs or scaffolds that are smaller than 10-20Kbp in the scaffolding process.

The haplotype phased assembly is much more fragmented, compared to both haploid assemblies, resulting in higher relative number of mate pairs mapping to different scaffolds. However, when looking at the “internal” mate pairs, i.e. where both mates map at least 10Kb away from the scaffold ends, we see remarkable consistency with less than 0.5% of the mates mapped to the wrong scaffold (see next section). Since for this analysis we only used mates mapping uniquely to the assembly, and there are two copies of the genome in the assembly, the total number of mapped mates is much lower.

Haplotype resolution.

To Illustrate the resolution of the haplotypes in Tur_tru_Illumina_phased_v1, we aligned it to Tur_tru_Illumina_hap_v1 using Nucmer tool. In Figure 2 we show the mummerplot of alignments of the phased assembly to the haploid one. The circles represent contig ends with lines joining them representing aligned sequence. The color indicates direction of the alignments. We display the alignments to an arbitrarily chosen scaffold314 of the haploid assembly. Only alignments longer than 5Kb are shown. Figure 2 shows that most of the “haploid” assembly aligns to two phased scaffolds, that is for each location on the x axis there are two corresponding alignments on the y axis. The regions that are covered by a single haplotype (rather than 2) are most probably homozygous regions of this genome. In cases where the homozygous region is long, it is more difficult to phase its heterozygous ends. Thus, in some cases, the homozygous regions are represented only once in the phased assembly (instead of twice). This is the cause for some of the single copy BUSCOs in the phased assembly. In our experience, this issue is more pronounced in mammalian phased assemblies due to the relatively lower heterozygosity level and the way it is distributed along the genome.

In haplotype phasing it is easy to phase small regions. For example, a single isolated SNP with no haplotype differences within 100 bp in both directions, can be trivially phased into two 201bp (or longer) contigs different by one base in the middle. It gets more difficult for larger contigs/scaffolds, where one must make sure that the contig/scaffold represents single haplotype and not a "mosaic" of haplotypes, that the SNPs and other bigger haplotype differences are correctly "phased". To do that we mapped the mate pairs from the 5-7Kb mate pair library to all phased scaffolds using Bowtie2 [28], and then examined the "internal" mate pairs where both reads in each pair mapped to the assembly, and one read mapped within 10Kb away from the ends of the scaffold. This would imply that the other mate must map to the same scaffold and not its haplotype, if haplotype phasing is done properly. If it does not, then it indicates an apparent mis-assembly or failure to phase haplotypes. By measuring the number of "properly" aligned internal mates, where both mates aligned to the same scaffold vs. "improper" internal mates where the mates aligned to different scaffolds, one can measure the efficacy of the haplotype phasing. There were 35,697,369 pairs where both mates mapped properly to the same scaffold, while only 169,244 mapped improperly, that is to two different scaffolds. The percentage of improperly mapping mate pairs is only 0.5%, indicating that haplotype resolution was done properly.

Synteny between human and dolphin. Dolphin is a mammal, and currently the best mammalian reference genome is the human genome. To understand similarities between dolphin and human on the DNA level, we aligned the Tur_tru_Illumina_hap_v1 assembly to the primary chromosomes of the current haploid human reference genome GRCh38 [26]. Since human and dolphin are fairly distant species, we did not expect to find long DNA sequence alignments but instead we were looking for synteny where relatively short DNA fragments of scaffolds align in the same order and orientation between the two assemblies. We used MUMmer4 package for producing the alignments using the default settings. The alignment mummerplot (Figure 3) shows a striking synteny between the dolphin assembled scaffolds and human chromosome 1, visible even on the large-scale chromosome plot (Figure 3a). No large-scale synteny to the other human chromosomes can be readily observed. The synteny observation is possible due to large scaffold sizes in Tur_tru_Illumina_hap_v1. In Figure 3b, we show 22 scaffolds that have 50% or more of their sequence in syntenic alignments. The syntenic alignments of these 22 scaffolds span nearly the entire human chromosome 1 sequence. The synteny is not a new finding, it was first identified by Bielec et al [22] and was later extended to many other placental mammals [23]. The Tur_tru_Illumina_hap_v1 assembly clearly illustrates and confirms the expected synteny.

Methods

Sample Collection and DNA Extraction: The sample for this study came from a female Atlantic bottlenose dolphin (Sample ID 04329), captive born at SeaWorld of Orlando, Orlando, Florida from wild male and female Atlantic bottlenose dolphins. The animal was 36 years old at blood collection with a healthy medical history. Blood was collected using Qiagen- PAXgene™ Blood DNA Tubes (Qiagen). High molecular weight genomic DNA was isolated using the Illumina- MasterPure™ DNA Purification Kit and subsequently quantified and qualified using Quant-iT™ dsDNA Kit and E-Gel™ EX Agarose Gel (ThermoFisher).

Collecting Sequence Data

Paired End (PE) libraries. We generated the 450bp and 800bp PE libraries using the Illumina TruSeq® PCR-free DNA Sample Prep kit. The protocol was slightly modified at fragmentation and double-size selection steps by adjusting the Covaris DNA shearing protocols and by empirically titrating the ratios of SPRI magnetic beads over

DNA to obtain insert sizes around 450bp and 800bp. We then evaluated the libraries for insert size and yield using Agilent Bioanalyzer and real-time qPCR assay, using Illumina DNA Standards and primer master mix qPCR kit (KAPA Biosystems, Roche), then normalized to 2nM prior to clustering and sequencing. Both the 450bp and 800 bp libraries were then denatured and diluted to 8pM and 12pM respectively. The 800bp PE library was clustered and sequenced on the HiSeq 2000, using the Illumina HiSeq Cluster and SBS v4 kits for PE 2x160bp reads. The 450bp PE library was clustered and sequenced on the HiSeq 2500 v2 Rapid Run mode using the HiSeq Rapid Cluster and SBS v2 kits for PE 2x250bp reads.

Mate Pair (MP) libraries. To maximize sequence diversity and genome coverage, three separate MP libraries were constructed corresponding to 2-5Kb, 5-7Kb and 7-10Kb insert sizes using the Nextera® MP Library Preparation Kit according to the manufacturer's instructions (Illumina). All three libraries were generated from a single input of 4ug of genomic DNA size-selected on a 0.8% E-gel (Invitrogen). Proper sizing of gel-extracted products was confirmed using the Bioanalyzer High Sensitivity chip (Agilent) and 600ng was subsequently used as input for circularization. Following library preparation, the Bioanalyzer was used to confirm library quality. Each of the three libraries were quantified by qPCR (KAPA Biosystems Library Quantification Kit, Roche), denatured and diluted to 200pM after size-adjustment according to Bioanalyzer results, and clustered on the cBot (Illumina) according to the manufacturer's instructions. 2x150bp of Illumina paired-end sequencing was performed on the HiSeq 4000 using the HiSeq 3000/4000 Cluster and SBS kits.

10x Chromium library. Genomic DNA quality was assessed by pulsed-field gel electrophoresis to determine suitability for 10X Chromium library preparation (10X Genomics). 1.125ng of input was used for library preparation according to the manufacturer's instructions without size-selection. Final library concentration was determined by qPCR (KAPA Library Quantification Kit, Roche) and size-adjusted according to Bioanalyzer DNA 100 chip (Agilent) results. 2x150bp of Illumina paired-end sequencing with an 8-base index read was performed on the HiSeq 4000 using the HiSeq 3000/4000 Cluster and SBS kits.

Genome Assembly

Genome assembly was conducted using the DeNovoMAGIC™ software platform (NRGene, Nes Ziona, Israel). This is a proprietary DeBruijn-graph-based assembler that was used to produce assemblies of several challenging plant genomes such as corn [1] and ancestral wheat Aegilops tauschii [3]. The following outlines design of the assembler, and steps of the assembly process.

Reads pre-processing. In the pre-processing step we first removed PCR duplicate reads, and trimmed Illumina adaptor AGATCGGAAGAGC and Nextera® linker (for MP library) sequences. We then merged the PE 450bp 2×250bp overlapping reads with minimal required overlap of 10bp to create stitched reads (SRs) using the approach similar to the one implemented in the Flash software [27].

Error correction. We scanned through all merged reads to detect and filter out reads with apparent sequencing errors by examining k-mers (k=24) in the reads and looking for low abundance k-mers. We have high coverage data (~450x), with each read yielding 127 (150-24+1) to 227 (250-24+1) K-mers. Thus average 24-mer coverage is at least 300x. 24-mers that only appear less than 10 times in the set of reads likely contain errors. We did not use the reads that contain these low abundance k-mers for building initial contigs.

Contig assembly. The first step of the assembly consists of building a De Bruijn graph (kmer=127 bp) of contigs from all filtered reads. Next, paired end and MP reads are used to find reliable paths in the graph between contigs for repeat resolving and contigs extension. 10x barcoded reads were mapped to

contigs to ensure that adjacent contigs were connected only when there is evidence that those contigs originate from a single stretch of genomic sequence (reads from the same two or more barcodes were mapped to the same contigs).

Split phased/un-phased assembly processes. Two parallel assemblies take place to complete the phased and un-phased assembly result. The phased assembly process utilizes the complete set of contigs. In the un-phased assembly process, the homologous contigs are identified and one of the homologs is filtered out, leaving a subset of the homozygous and one of the homologous contigs in heterozygous regions. The linking information of both homologous contigs is kept through the assembly process of the un-phased assembly, usually enabling longer un-phased scaffolds.

Scaffolding. All the following steps are done in parallel for both the phased and un-phased assemblies. Contigs were linked into scaffolds with PE and MP information, estimating gaps between the contigs according to the distance of PE and MP links. In addition, for the phased assembly, 10x data were used to validate and support correct phasing during scaffolding.

Gap filling. A final gap fill step used PE and MP links and De Bruijn graph information to locally construct a unique path through the graph connecting the gap edges. The path was used to close the gap if it was unique and its length was consistent with the gap size estimate.

Scaffold split/merge. We used 10x barcoded reads to refine and merge scaffolds. All barcoded 10x reads were mapped to the assembled scaffolds. Clusters of reads with the same barcode mapped to adjacent contigs in the scaffolds were identified to be part of a single long molecule. Next, each scaffold was scanned with a 20kb length window to ensure that the number of distinct clusters that cover the entire window (indicating a support for this 20kb connection by several long molecules) is statistically significant with respect to the number of clusters that span the left and the right edge of the window. If there was a statistically significant disagreement in the coverage by the clusters over the window, we broke the scaffold at the two edges of the window. Finally, the barcodes that were mapped to the scaffold edges (first and last 20kb sequences) were compared to generate a graph of scaffolds. The scaffolds are nodes and the edges are links connecting nodes with more than two common barcodes on the ends. We broke the links to the nodes that had more than two links and output the resulting linear paths in the scaffold graph as final scaffolds.

Summary

We show that Tur_tru_Illumina_hap_v1 is more complete and accurate compared to the current best reference Tur_tru v1, based on the amount and composition of sequence, the consistency of the MP alignments to the assembled scaffolds, and on the analysis of conserved single-copy mammalian orthologs. The additional 12.5% of sequence data identified and assembled here was found to contain 165 additional BUSCO alignments as compared to the latest published assembly Tur_tru v1. The large scaffolds represented by Tur_tru_Illumina_hap_v1 enabled and confirmed expected synteny to human chromsome 1. The phased de novo assembly Tur_tru_Illumina_phased_v1 is of the first publicly available and it provides the community with novel ways to explore the heterozygous nature of the dolphin genome. These findings illustrate the impact of improved sample preparation and improved de novo assembly methods on progress toward more complete and accurate reference quality genomes. Better quality assemblies will improve our understanding of gene structure, function and evolution in mammalian species.

Table 1. Summary of the sequencing data collected to create Tur_tru_Illumina_hap_v1 and Tur_tru_Illumina_phased_v1.

Library type	Read Length	Insert Size	Genomic Coverage
**PCR-free**	2x250bp	450bp	101x
**PCR-free**	2x160bp	800bp	123x
**Mate-Pair**	2x150bp	2-4Kbp (peak 4.2Kbp)	37x
**Mate-Pair**	2x150bp	5-7Kbp (peak 6.0Kbp)	61x
**Mate-Pair**	2x150bp	8-10Kbp (peak 9.9Kbp)	58x
**10X Chromium**	2x150bp	-	70x

Table 2. Comparison of quantitative statistics for different assemblies of the bottlenose dolphin. The total sequence listed excludes Ns (ambiguous nucleotides). Ns were also squeezed out from the scaffolds for N50 computations. We used genome size of 2,383,130,043bp equal to the total amount of sequence in the scaffolds of the bigger haploid assembly, for comparison of the N50 contig and scaffold sizes between the two assemblies. The Tur_tru_Illumina_hap_v1 and Tur_tru v1 assemblies have comparable scaffold N50 sizes, and Tur_tru v1 has bigger contigs. The Tur_tru_Illumina_hap_v1 assembly has more sequence and our BUSCO analysis (Table 3) shows that it is likely more complete. The N50 comparisons to the haplotype-resolved Tur_tru_Illumina_phased_v1 assembly are shown for completeness, computed with 2x genome size (2*2,383,130,043 = 4,766,260,086bp).

	Tur_tru v1	Tur_tru_Illumina_hap_v1	Tur_tru_Illumina_phased_v1
**Total sequence**	2,120,283,832	2,383,130,043	4,678,362,582
**# of scaffolds**	2647	481	98,209
**Longest scaffold**	96,299,184	83,924,496	10,429,594
**Scaffold N50**	23,564,561	26,997,441	777,432
**Scaffold L50**	26	30	1,509
**# of contigs**	116,650	139,544	355,974
**Longest contig**	403,070	320,783	298,006
**Contig N50**	37,749	30,985	25,997
**Contig L50**	17,321	23,199	53,738
**GC content**	40.85	41.25	41.95

Table 3. Comparison of BUSCO 3.0.2 Mammalia single copy orthologs among the three Dolphin assemblies. The table shows that the Tur_tru_Illumina_hap_v1 assembly is more complete, with 110 fewer missing single-copy orthologs compared to the Tru_tru v1 assembly. The Tur_tru_Illumina_hap_v1 assembly has 43 extra duplicated orthologs, which possibly points to incomplete filtering of redundant haplotypes. While Tur_tru v1 assembly has bigger contigs, the Tur_tru_Illumina_hap_v1 assembly has many fewer fragmented BUSCOs. The haplotype-resolved Tur_tru_Illumina_phased_v1 assembly is less contiguous and less complete. As expected, more than half of the complete BUSCOs are duplicated, corresponding to the two resolved haplotypes.

BUSCOs	**Tur_tru v1**	**Tur_tru_Illumina_hap_v1**	**Tur_tru_Illumina_phased_v1**
Complete	3,647	3,837	3,537
Complete single-copy	3,614	3,760	1,310
Complete duplicated	33	77	2,227
Fragmented	187	107	301
Missing	270	160	266
Total	4,104	4,104	4,104

Table 4. Comparison of the number of mate pairs (MPs) from 5-7Kbp library uniquely aligned to Tur_tru_Illumina_hap_v1, Tur_tru_Illumina_phased_v1 and Tur_tru v1 assemblies. The alignments were done with Bowtie2. Only the reads that mapped **uniquely** were used for this computation, thus the number of MPs uniquely mapping to haplotype resolved assembly is much smaller. Same scaffold means that both mates mapped to the same scaffold; happy mates aligned in the correct orientation with mate distance within 5 standard deviations from the mean; misoriented mates aligned in wrong orientation; long mates aligned with the distance between the mates exceeding 5 standard deviations; short mates aligned with the distance of less than 1000bp. Same scaffold mate ALL is the total number of all mate pairs where both mates aligned to the same scaffold.

	Tur_tru_Illumina_hap_v1	Tur_tru_Illumina_phased_v1	Tur_tru v1
**Same scaffold mate ALL**	228,285,253	73,514,546	219,277,963
**Same scaffold happy**	125,224,663	37,248,420	117,942,390
**Same scaffold misoriented**	158,547	51,533	1,164,118
**Same scaffold long**	82,948	9,183	274,458
**Same scaffold short**	102,819,095	36,205,410	99,896,997
**Mates aligned to different scaffolds**	5,629,393	7,438,471	5,576,802
**One mate in the pair aligned**	61,284,891	26,228,913	68,707,025

Availability of data

The dolphin assembly Tur_tru_Illumina_hap_v1 has been deposited at NCBI under BioProject PRJNA476133, accession QMGA00000000. The dolphin assembly Tur_tru_Illumina_phased_v1 has been deposited at NCBI under BioProject PRJNA478376, accession QUXD00000000. Both assemblies are also available on the public FTP site ftp://ftp.ccb.jhu.edu/pub/alekseyz/Tur_tru_Illumina_v1/. All data are also available from the *GigaScience* GigaDB repository [29].

Competing interest

KV and CTL were both full-time employees of Illumina at the time this work was completed. GBZ, KB, and TB are employees of NRGene, a company providing software analysis tools for de novo assembly.

Author contributions

KVM, CTL and MMV designed the project. KVM, CTL and AZ wrote the manuscript. GBZ, TB and KB generated genome assemblies. AZ conducted validation, MP consistency analysis, human chromosome 1 and BUSCO analyses, and submitted the genomes to NCBI; JSL provided the blood sample; JL, AN, MR, MG, EJ, and BS processed samples, generated sequencing and completed quality checks on sequence data; and all authors contributed to editing the manuscript.

Abbreviations

BUSCO: benchmarking universal single-copy orthologs; Kbp: kilobase pairs; MP: mate pairs; Ns: ambiguous nucleotides; PE: paired end; SR: stitched reads.

Acknowledgement

This project was sponsored by Illumina, Inc. and NRGene. The authors acknowledge the support of the following people: Tristan Orpin and Matt Posard previously at Illumina, Inc.; Mark Van Oene, Feng Chen, Christine Ching, Shu Boles, Zheng Xu, Joey Flores, Kan Nobuta, Brad Sickler, Courtney McCormick, Christopher Haynes, Jennifer Bernet, and Nathalie Mouttham from Illumina, Inc.; Melissa Katigbak and Shara Fisler from Ocean Discovery Institute; Todd Schmitt from SeaWorld; Edwin Hauw, Deanna Church, and John Stuelpnagel from 10x Genomics.

410

Figure 1. Venn diagram of BUSCOs present in two dolphin assemblies. Out of 4,104 BUSCOs in mammalia set, 105 are missing from both assemblies. Our assembly has 165 BUSCOs not present in Tur_tru v1 and Tur_tru v1 has 55 BUSCOs that are not present in our assembly.

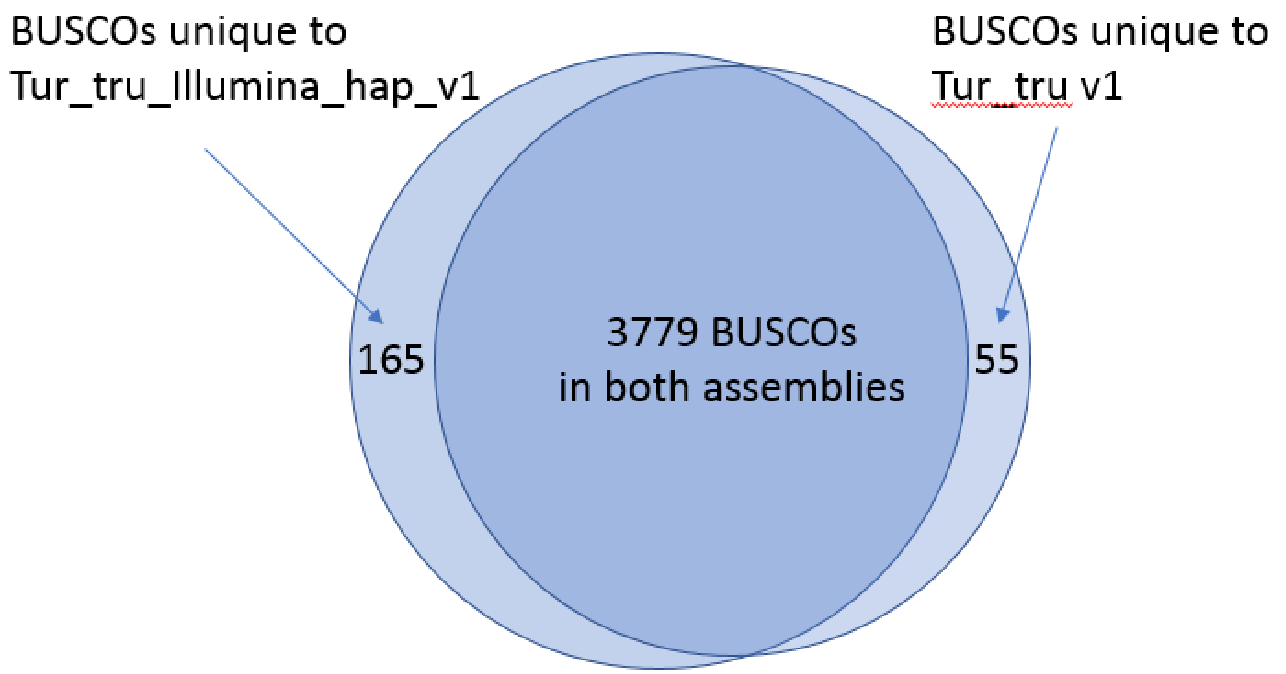

Figure 2. An example mummerplot of the alignments of the phased assembly to the “haploid” one, spanning about 15Mbp of sequence of scaffold314. The circles represent contig ends with lines joining them representing aligned sequence. The color indicates direction of the alignment with red and blue forward and reverse respectively. We show that for most locations on the x-axis (haploid assembly coordinate) there are two alignments on the y-axis corresponding to the two phased haplotypes. The small number of regions with a single contig aligning represent long homozygous regions of the genome that we were unable to phase.

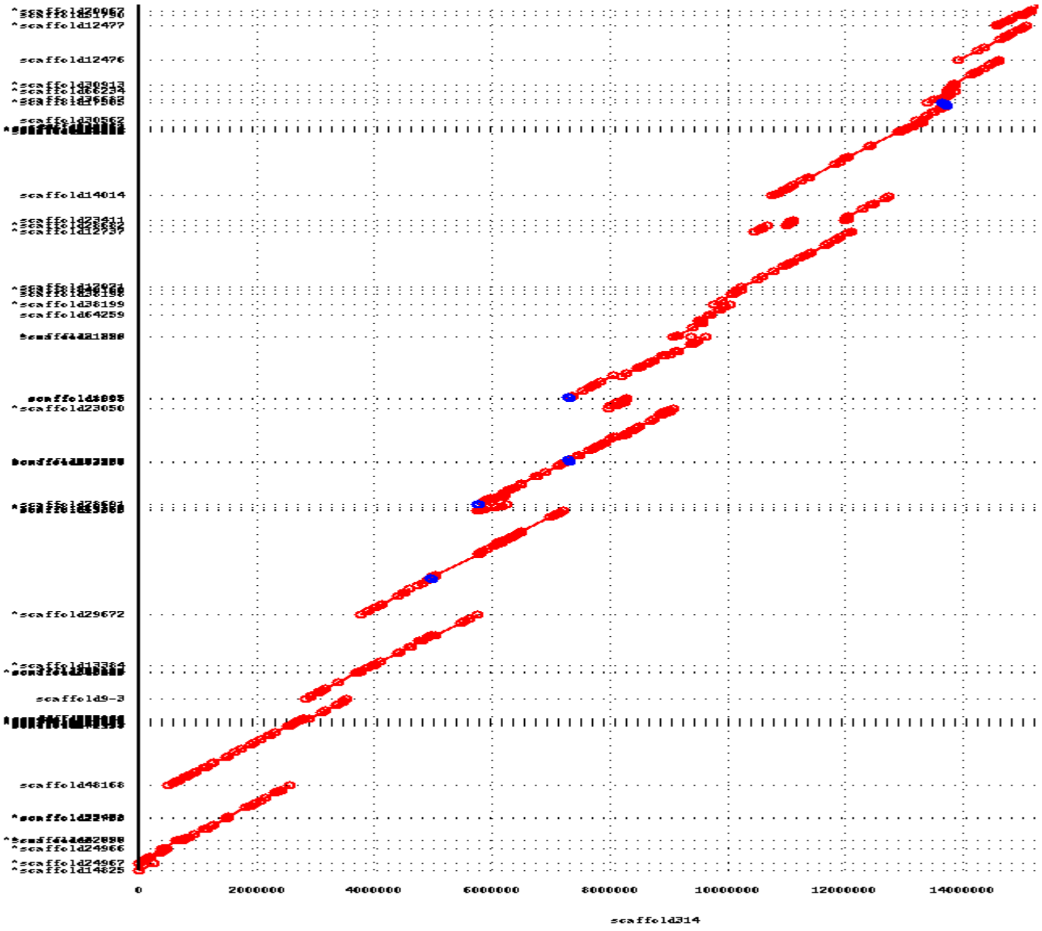

Figure 3. This figure shows the alignment of the Tur_tru_Illumina_hap_v1 assembly to the human GRCH38 reference (primary chromosomes only). Each dot represents an alignment with red indicating forward direction and blue indicating reverse direction. Human reference coordinates are on the X axis and Tur_tru_Illumina_hap_v1 assembly alignment coordinates are on the Y axis. Panel (a) shows alignment of the entire assembly to the human reference with alignments to human chromosome 1 highlighted by the black box. One can clearly see the synteny that is present between the dolphin scaffolds and human chromosome 1. No other human chromosome shows clear synteny. Dolphin scaffolds with syntenic alignments spanning over 50% of the scaffold were extracted. Alignments only to human chromosome 1 are presented in panel (b).

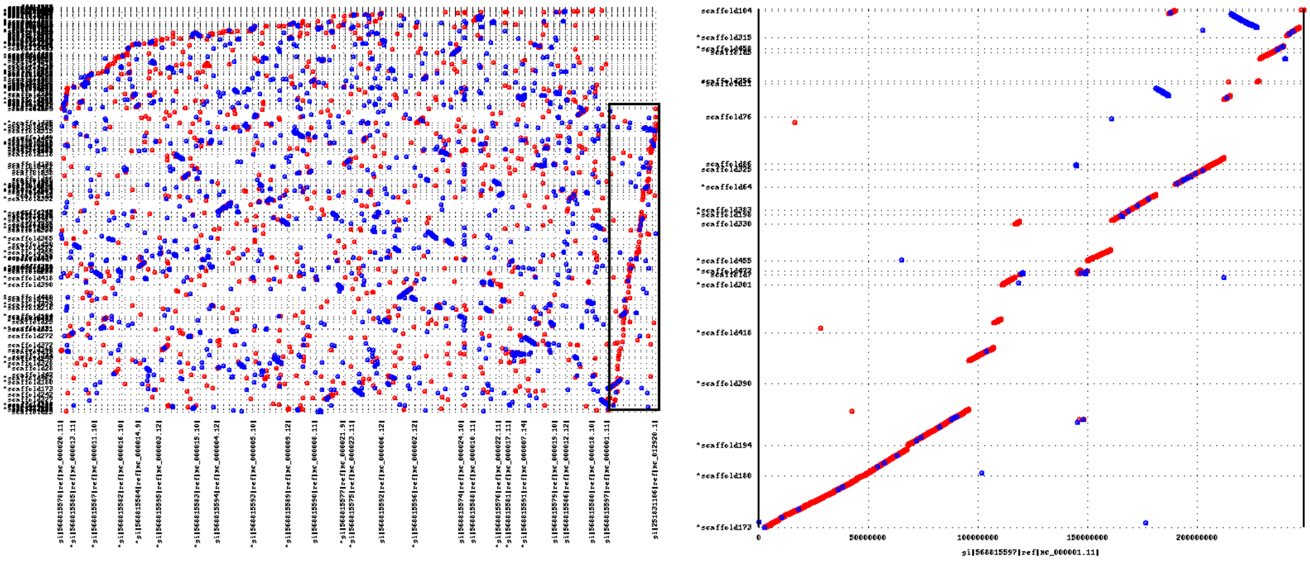

(a) (b)

References

1. Hirsch CN, Hirsch CD, Brohammer AB et al. Draft Assembly of Elite Inbred Line PH207 Provides Insights into Genomic and Transcriptome Diversity in Maize. Plant Cell 2016;28:2700-2714

2. Avni R, Nave M, Barad O et al. Wild emmer genome architecture and diversity elucidate wheat evolution and domestication. Science 2017;357:93-97

3. Luo MC, Gu YQ, Puiu D et al. Genome sequence of the progenitor of the wheat D genome Aegilops tauschii. Nature 2017;551:498-502

4. Zimin A, Stevens KA, Crepeau MW et al. An improved assembly of the loblolly pine mega-genome using long-read single-molecule sequencing. GigaScience 2017;6:1-4

5. Genome 10K Community of Scientists. Genome 10K: A Proposal to Obtain Whole-Genome Sequence for 10000 Vertebrate Species. Journal of Heredity 2009;100(6):659-674

6. Koepfli K, Paten B, Antunes A et al. The Genome 10K Project: A Way Forward Further. Annual Review of Animal Biosciences 2015;3:57-111

7. Lewin HA, Robinson GE, Kress WJ et al. Earth BioGenome Project: Sequencing life for the future of life. Proceedings of the National Academy of Sciences 2018;115(17):4325-4333

8. Mohr DW, Naguib A, Weisenfeld N et al. Improved *de novo* Genome Assembly: Linked-Read Sequencing Combined with Optical Mapping Produce a High Quality Mammalian Genome at Relatively Low Cost. bioRxiv 2017;128348

9. Armstrong EE, Taylor RW, Prost S et al. Entering the era of conservation genomics: Cost-effective assembly of the African wild dog genome using linked long reads. Gigascience. 2018 Oct 22. doi: 10.1093/gigascience/giy124.

10. Hammond PS, Bearzi G, Bjørge A et al. *Tursiops truncatus*. The IUCN Red List of Threatened Species 2012:e.T22563A17347397. http://dx.doi.org/10.2305/IUCN.UK.2012.RLTS.T22563A17347397.en.

11. Rosel PE, Hancock-Hanser BL, Archer FI et al. Examining metrics and magnitudes of molecular genetic differentiation used to delimit cetacean subspecies based on mitochondrial DNA control region sequence. Special Issue: Delimiting subspecies using primarily genetic data. Marine Mammal Science 2017;33(S1):76-100

12. McGowen MR, Grossman LI, Wildman DE. Dolphin genome provides evidence for adaptive evolution of nervous system genes and a molecular rate slowdown. Proc. R. Soc. B 2012;279:3643–3651

13. Venn-Watson S, Carlin K, Ridgway S. Dolphins as animal models for type 2 diabetes: sustained, post-prandial hyperglycemia and hyperinsulinemia. Gen Comp Endocrinol 2011; 170(1):193-9

14. Venn-Watson S, Smith CR, Stevenson S et al. Blood-Based Indicators of Insulin Resistance and Metabolic Syndrome in Bottlenose Dolphins (*Tursiops truncatus*). Front Endocrinol (Lausanne) 2013;4:136

15. Venn-Watson S. Dolphins as animal models for type 2 diabetes: sustained, post-prandial hyperglycemia and hyperinsulineia. Frontiers in Endocrinology 2014; 227

16. Neely BA, Debra L. Ellisor DL et al. Proteomics as a metrological tool to evaluate genome annotation accuracy following de novo genome assembly: a case study using the Atlantic bottlenose dolphin (*Tursiops truncatus*). bioRxiv 2018;254250

17. Sobolesky P, Parry C, Boxall B et al. Proteomic analysis of non-depleted serum proteins from bottlenose dolphins uncovers a high vanin-1 phenotype. Scientific Reports 2016;26(6):33879

18. Venn-Watson S, Smith CR, Gomez F et al. Physiology of aging among healthy, older bottlenose dolphins (*Tursiops truncatus*): comparisons with aging humans. J Comp Physiol B. 2011;181(5):667-80

19. Lindblad-Toh K, Garber M , Zuk O et al. A high-resolution map of human evolutionary constraint using 29 mammals. Nature 2011;478:10530

20. Foote AD, Liu Y, Thomas GWC et al. Convergent evolution of the genomes of marine mammals.2014. Nature Genetics 2014;47,3: 272-275

21. Marks P, Garcia S, Alvaro Martinez A et al. Resolving the Full Spectrum of Human Genome Variation using Linked-Reads. bioRxiv 2018;230946

22. Bielec PE, Gallagher DS, Womack JE et al. Homologies between human and dolphin chromosomes detected by heterologous chromosome painting. Cytogenetic and Genome Research 1998;81(1):18-25

23. Murphy WJ, Frönicke L, O'Brien SJ et al. The origin of human chromosome 1 and its homologs in placental mammals. Genome research. 2003;13(8):1880-8

24. Marçais G, Delcher AL, Phillippy AM et al. MUMmer4: A fast and versatile genome alignment system. PLOS 2018;1005944

25. Putnam NH, O'Connell BL, Stites JC et al. Chromosome-scale shotgun assembly using an in vitro method for long-range linkage. Genome Research 2016;26(3):342-50

26. Schneider VA, Graves-Lindsay T, Howe K et al. Evaluation of GRCh38 and de novo haploid genome assemblies demonstrates the enduring quality of the reference assembly. Genome research 2017;27(5):849-64

27. Magoč T, Salzberg SL. FLASH: fast length adjustment of short reads to improve genome assemblies. Bioinformatics 2011;27(21):2957-63

28. Langmead B, Salzberg SL. Fast gapped-read alignment with Bowtie 2. Nature methods. 2012;9(4):357-359. doi:10.1038/nmeth.1923.

29. Martinez KV; Lawley CT; Vergara MM; Ben-Zvi G; Biniashvili T; Baruch K; Leger JS; Le J; Natarajan A; Rivera M; Guillergan M; Jaeger E; Steffy B; Zimin AV (2018): Supporting data for "New de novo assembly of the Atlantic bottlenose dolphin (*Tursiops truncatus*) improves genome completeness and provides haplotype phasing" GigaScience Database. http://dx.doi.org/10.5524/100546

Manuscript
Click here to access/download;Manuscript;GIGAScience_v26_final.docx
Click here to view linked References

New de novo assembly of the Atlantic bottlenose dolphin (*Tursiops truncatus*) improves genome completeness and provides haplotype phasing.

Karine A. Martinez-Viaud *[1], Cindy Taylor Lawley*[2,6], Milmer Martinez Vergara*[3], Gil Ben-Zvi[4], Tammy Biniashvili[4], Kobi Baruch[4], Judy St. Leger[5], Jennie Le[1], Aparna Natarajan [1], Marlem Rivera[1,6], Marbie Guillergan[1], Erich Jaeger [1], Brian Steffy [1] and Aleksey Zimin[7]

1. Illumina, Inc, San Diego, CA 92122, USA
2. GinkgoFish LLC, Carson City, NV 89703
3. Plant with Purpose, San Diego, CA 92117, USA
4. NRGene, Ness-Ziona, 7403649, Israel
5. SeaWorld San Diego, San Diego, CA 92109, USA
6. Ocean Discovery Institute, San Diego, CA 92109 USA
7. Johns Hopkins University, Baltimore, MD 21205, USA
*Authors contributed equally to the project
**Corresponding authors: kviaud@illumina.com and alekseyz@jhu.edu

Karine A. Martinez-Viaud kviaud@illumina.com
Cindy Taylor Lawley cindylawleyphd@gmail.com
Milmer Martinez Vergara milmer@plantwithpurpose.org
Gil Ben-Zvi gil@nrgene.com
Tammy Biniashvili tammy@nrgene.com
Kobi Baruch kobi@nrgene.com
Judy St. Leger Judy.St.Leger@SeaWorld.com
Jennie Le jle@illumina.com
Aparna Natarajan anatarajan1@illumina.com
Marlem Rivera mrivera@illumina.com
Marbie Guillergan mguillergan@illumina.com
Erich Jaeger ejaeger@illumina.com
Brian Steffy bsteffy@illumina.com
Aleksey Zimin alekseyz@jhu.edu

Abstract

High quality genomes are essential to resolve challenges in breeding, comparative biology, medicine and conservation planning. New library preparation techniques along with better assembly algorithms result in continued improvements in assemblies for non-model organisms, moving them toward reference quality genomes. We report on the latest genome assembly of the Atlantic bottlenose dolphin leveraging Illumina sequencing data coupled with a combination of several library preparation techniques. These include Linked-Reads (Chromium, 10x Genomics), mate pairs, long insert paired ends and standard paired ends. Data were assembled with the commercial DeNovoMAGIC™ assembly software resulting in two assemblies, a traditional "haploid" assembly (Tur_tru_Illumina_hap_v1) that is a mosaic of the two parental haplotypes and a phased assembly (Tur_tru_Illumina_phased_v1) where each scaffold has sequence from a single homologous chromosome. We show that Tur_tru_Illumina_hap_v1 is more complete and accurate compared to the current best reference based on the amount and composition of sequence, the consistency of the mate pair alignments to the assembled scaffolds, and on the analysis of conserved single-copy mammalian orthologs. The phased de novo assembly Tur_tru_Illumina_phased_v1 is the first publicly available for this species and provides the community with novel and accurate ways to explore the heterozygous nature of the dolphin genome.

Keywords: de novo genome assembly, bottlenose dolphin, Tursiops truncatus, 10x Genomics, DeNovoMAGIC™, Illumina

Introduction

Technical advances in the past decade have reduced sequencing costs and improved access to sequencing data. Subsequent improvements in DNA extraction, preparation, and assembly algorithms facilitate low cost accurate de novo genome assemblies. Such assemblies are essential for constructing haplotype diversity databases for breeding, comparative biology, medicine and conservation planning. Even highly complex genomes now benefit from higher contiguity and improved protein coding coverage [1-4]. Consortium efforts to catalogue biodiversity of pivotal species of comparative evolutionary significance will continue to drive novel low-cost approaches toward reference quality assemblies with chromosome level resolution [5-7]. Here we use a combination of methods to drive improvements in assembly structure for the Atlantic bottlenose dolphin (*Tursiops truncatus*). This genome assembly, like that of the Hawaiian Monk seal and African wild dog, is being published with the goal to facilitate research on comparative genomics, provide structure for cataloging biodiversity and ultimately support decisions around species conservation and management [8, 9].

The bottlenose dolphin is one of the most widely studied marine mammals, however the taxonomy of the *Tursiops* genus remains unresolved. Numerous species designations have been suggested but not adopted due to a lack of resolution afforded by available data [10]. Even with new molecular genetic markers, we have reached a limitation on resolution from genetic data available to delineate species, subspecies and populations [11]. To usher this species into the era of genomics, a high-quality reference genome is essential. It provides structure to catalogue diversity within and between species at the whole genome level. In addition, the parallel molecular trajectory between dolphin and other mammalian species [12] makes the bottlenose dolphin a useful model to understand aspects of human health such as metabolic processes/diabetes [13-15], proteomics [16, 17] and aging [18].

A preliminary dolphin genome was first submitted to NCBI (TurTru1.0; GCA_000151865.1) using low coverage (2.82X) Sanger sequencing for the purpose of cross-species comparison [12, 19, 20]. Subsequent improvements were achieved through the addition of 30X Illumina short read data and 3.5X 454 data (Ttru_1.4; GCA_000151865.3). A much more complete genome was submitted in 2016 leveraging improvements in library preparation and assembly methods (Meraculous v. 2.2.2.5 and HiRise v. 1.3.0-116-gf50c3ce; Dovetail, Inc) with 114X coverage of Illumina HiSeq data prepared with proximity ligation Hi-C protocol (Tur_tru v1; GCA_001922835.1; [16]).

With the collection of data from multiple sources including Linked-Reads (Chromium, 10x Genomics; [21]), mate pairs (MP), long insert paired ends and standard paired ends, and the DeNovoMAGIC assembly tool (NRGene, Ness-Ziona, Israel), we provide an improved haploid reference quality dolphin genome assembly as well as the first haplotype phased diploid assembly. We refer to our unphased assembly as Tur_tru_Illumina_hap_v1 and to the phased assembly as Tur_tru_Illumina_phased_v1. Using Tur_tru v1 for comparison, our assembly shows increased contiguity and completeness with high consistency to the MP data and orthologous mammalian protein alignments. Additionally, by aligning Tur_tru_Illumina_hap_v1 to the Human reference genome, we illustrate the synteny of the dolphin scaffolds to human chromosome 1 [22, 23].

Results

Coverage. We generated sequence data for a total coverage of approximately 450X, the majority from PCR Free and Chromium 10X Genomics Linked-Read libraries (Table 1). Coverage was computed using 2.4Gbp estimated genome size. Genome assembly was conducted using DeNovoMAGIC^{TM} software (NRGene, Ness-Ziona, Israel). More detail about the library preparation and the assembly process are found in the Methods section.

Haploid and diploid assemblies. We report on two assemblies in this manuscript, one traditional haploid consensus assembly Tur_tru_Illumina_hap_v1 that represents a mosaic of the maternal and paternal haplotypes, and the other haplotype-phased (i.e., diploid) assembly where each scaffold represents sequence corresponding to a single haplotype, Tur_tru_Illumina_phased_v1. The quantitative statistics for both assemblies are listed in Table 2. The phased or diploid genome assembly was made possible using Illumina sequencing data by leveraging the combination of library prep methods including Linked-Reads, is a significant advance and will provide the community with a powerful genomic tool for the downstream analysis in the context of the true heterozygous dolphin genome.

Genome assembly comparison. Both assemblies were compared to the best available assembly Tur_tru v1 (NCBI accession GCA_001922835.1; [16]). We did not use the Ttru_1.4 assembly (NCBI accession GCA_000151865.3) because the contiguity statistics of the Ttru_1.4 are vastly inferior to the Tur_tru v1 with a contig N50 3 times smaller than Tur_tru v1 and scaffold N50 over 200 times smaller.

The statistics for the Tur_tru_Illumina_hap_v1 assembly show bigger scaffolds but slightly smaller contigs with about 13% more sequence in the scaffolds compared to Tur_tru v1 (Table 2). More sequence does not necessarily make for a better assembly considering that the extra sequence may be duplicated haplotypes or contaminants that do not belong to the original organism. To characterize the extra sequence, we first aligned the Tur_tru_Illumina_hap_v1 to the Tur_tru v1 assembly using the Nucmer aligner which is part of MUMmer4 package [24]. We used default settings for generating the alignments. We then analyzed the alignments using the dnadiff package included with MUMmer4. 87.5% of Tur_tru_Illumina_hap_v1 sequence aligned to 97.9% of Tur_tru v1. This shows that 12.5% of Tur_tru_Illumina_hap_v1 had no alignments to Tur_tru v1, while only 2.1% of Tur_tru v1 had no alignments to Tur_tru_Illumina_hap_v1. Therefore, there are 301Mbp of extra novel sequence in our new assembly Tur_tru_Illumina_hap_v1. We then used the BUSCO tool to show that the extra sequence is meaningful (Table 3). Tur_tru_Illumina_hap_v1 had 160 missing BUSCOs, compared to 270 missing in Tur_tru v1. The number of duplicated BUSCOs was higher by only 34 in our assembly compared to Tur_tru v1. This suggests that most of the extra sequence in Tur_tru_Illumina_hap_v1 is not contamination or redundant sequence, and likely contains useful coding information. There were 105 BUSCOS missing from both assemblies. We examined the locations of the 165 BUSCOs that are only found in the Tur_tru_Illumina_hap_v1 and all of them fully or partially aligned to locations in the sequences that were missing in Tur_tru v1 assembly. Figure 1 shows the Venn diagram of BUSCOs aligned to both assemblies, showing that there are 165 BUSCOS that are only present in Tur_tru_Illumina_hap_v1 and 55 that are only present in Tur_tru v1, with 3779 present in both assemblies. The haplotype-resolved assembly is more fragmented and it is missing 266 BUSCOs. As expected most of the complete BUSCOs that were found (3537) are duplicated 2227), since they are found in different haplotypes.

Assembly validation through MP consistency. Since both Tur_tru v1 and Tur_tru_Illumina_hap_v1 reference the same species, we expect few rearrangements between the assemblies. To examine this, we compared the absolute and relative correctness of the scaffolds of Tur_tru_Illumina_hap_v1 assembly by aligning the Illumina data from the 5-7Kbp MP library to the scaffolds of

Tur_tru_Illumina_hap_v1, Tur_tru_Illumina_phased_v1,and Tur_tru v1 assemblies using the Bowtie2 tool [28]. We chose this library because it contained the largest number of valid 5-7Kbp mate pairs. We then used only high quality uniquely aligning mated reads (both mates had to align uniquely with quality score 42 in the SAM file) and classified the alignments of the MPs into the following categories (Table 4):

1. **Same scaffold happy** – number of MPs where both mates aligned to the same scaffold in the correct orientation with mate separation within 3 standard deviations of the library mean
2. **Same scaffold short** -- number of MPs where both mates aligned to the same scaffold in the opposite orientation with mate separation of less than 1000bp; these MPs are not indicative of scaffolding misassemblies, they are simply a byproduct of the mate pair library preparation process as they are MPs that are missing the circularization junction site between the mates
3. **Same scaffold long** – number of MPs where both mates aligned to the same scaffold in the correct orientation, but the mate separation exceeded three standard deviations of the library mean
4. **Same scaffold misoriented** -- number of MPs where both mates aligned to the same scaffold in the opposite orientation with mate separation of more than 1000bp
5. **Mates aligned to different scaffolds** – number of MPs where the two mates aligned to different scaffolds
6. **Only one mate in the pair aligned** – number of MPs where only one read aligned to the assembly.

"Same scaffold ALL" category in table 4 is the sum of all mates in categories 1 to 4, it is listed for completeness.
Comparing Tur_tru_Illumina_hap_v1 with Tur_tru v1, the total number of reads uniquely aligning to both "haploid" assemblies is very similar: about 295.2M reads aligned to Tur_tru_Illumina_hap_v1 vs. about 293.6M reads aligned to Tur_tru v1. The total number of mate pairs aligning to the same scaffold is larger for Tur_tru_Illumina_hap_v1. Of the mate pairs aligning to the same scaffold, the number of mate pairs in the "Same scaffold happy" category is very similar between the two assemblies. The differences that stand out are the much larger (7.3 times more) number of mates that aligned to the same scaffold in the wrong orientation and the much larger (3.3 times more) number of the same scaffold long pairs in Tur_tru v1 compared to Tur_tru_Illumina_hap_v1 (Table 4). Of course, some level of discrepancy is expected, because the two assemblies represent two different individuals with unknown level of structural variation between them. However, in concert, the two different categories may also suggest a possibility of a relatively higher number of locally mis-ordered or misoriented contigs in the scaffolds of Tur_tru v1 assembly. This may be due to the scaffolding process used to create Tur_tru v1 assembly. The assembly was created with the HiRise assembler [25] using proximity ligation Hi-C data for scaffolding. The proximity ligation data provide MPs of all possible sizes, however, the MP distances and mate orientations are unknown. Since there are more shorter pairs than longer pairs due to the 3D structure of the DNA – it is much more likely to ligate parts of DNA that are closer to each other than the ones that are far apart. This property enables one to use these data for scaffolding. By mapping the pairs to the assembled scaffolds, one can measure how the distance between the mates in a pair varies with the number of pairs whose ends map to the same location in the assembly. However, the dependence is weak on the short end, meaning that the number of pairs of about 10Kbp in length is not much different from the number of links of 12-13Kbp in length. This frequently results in mis-orientations and shuffling of scaffold positions for contigs or scaffolds that are smaller than 10-20Kbp in the scaffolding process.

The haplotype phased assembly is much more fragmented, compared to both haploid assemblies, resulting in higher relative number of mate pairs mapping to different scaffolds. However, when looking at the "internal" mate pairs, i.e. where both mates map at least 10Kb away from the scaffold ends, we see remarkable consistency with less than 0.5% of the mates mapped to the wrong scaffold (see next section). Since for this analysis we only used mates mapping uniquely to the assembly, and there are two copies of the genome in the assembly, the total number of mapped mates is much lower.

Haplotype resolution.
To Illustrate the resolution of the haplotypes in Tur_tru_Illumina_phased_v1, we aligned it to Tur_tru_Illumina_hap_v1 using Nucmer tool. In Figure 2 we show the mummerplot of alignments of the phased assembly to the haploid one. The circles represent contig ends with lines joining them representing aligned sequence. The color indicates direction of the alignments. We display the alignments to an arbitrarily chosen scaffold314 of the haploid assembly. Only alignments longer than 5Kb are shown. Figure 2 shows that most of the "haploid" assembly aligns to two phased scaffolds, that is for each location on the x axis there are two corresponding alignments on the y axis. The regions that are covered by a single haplotype (rather than 2) are most probably homozygous regions of this genome. In cases where the homozygous region is long, it is more difficult to phase its heterozygous ends. Thus, in some cases, the homozygous regions are represented only once in the phased assembly (instead of twice). This is the cause for some of the single copy BUSCOs in the phased assembly. In our experience, this issue is more pronounced in mammalian phased assemblies due to the relatively lower heterozygosity level and the way it is distributed along the genome.

In haplotype phasing it is easy to phase small regions. For example, a single isolated SNP with no haplotype differences within 100 bp in both directions, can be trivially phased into two 201bp (or longer) contigs different by one base in the middle. It gets more difficult for larger contigs/scaffolds, where one must make sure that the contig/scaffold represents single haplotype and not a "mosaic" of haplotypes, that the SNPs and other bigger haplotype differences are correctly "phased". To do that we mapped the mate pairs from the 5-7Kb mate pair library to all phased scaffolds using Bowtie2 [28], and then examined the "internal" mate pairs where both reads in each pair mapped to the assembly, and one read mapped within 10Kb away from the ends of the scaffold. This would imply that the other mate must map to the same scaffold and not its haplotype, if haplotype phasing is done properly. If it does not, then it indicates an apparent mis-assembly or failure to phase haplotypes. By measuring the number of "properly" aligned internal mates, where both mates aligned to the same scaffold vs. "improper" internal mates where the mates aligned to different scaffolds, one can measure the efficacy of the haplotype phasing. There were 35,697,369 pairs where both mates mapped properly to the same scaffold, while only 169,244 mapped improperly, that is to two different scaffolds. The percentage of improperly mapping mate pairs is only 0.5%, indicating that haplotype resolution was done properly.

Synteny between human and dolphin. Dolphin is a mammal, and currently the best mammalian reference genome is the human genome. To understand similarities between dolphin and human on the DNA level, we aligned the Tur_tru_Illumina_hap_v1 assembly to the primary chromosomes of the current haploid human reference genome GRCh38 [26]. Since human and dolphin are fairly distant species, we did not expect to find long DNA sequence alignments but instead we were looking for synteny where relatively short DNA fragments of scaffolds align in the same order and orientation between the two assemblies. We used MUMmer4 package for producing the alignments using the default settings. The alignment mummerplot (Figure 3) shows a striking synteny between the dolphin assembled scaffolds and human chromosome 1, visible even on the large-scale chromosome plot (Figure 3a). No large-scale synteny to the other human chromosomes can be readily observed. The synteny observation is possible due to large scaffold sizes in Tur_tru_Illumina_hap_v1. In Figure 3b, we show 22 scaffolds that have 50% or more of their sequence in syntenic alignments. The syntenic alignments of these 22 scaffolds span nearly the entire human chromosome 1 sequence. The synteny is not a new finding, it was first identified by Bielec et al [22] and was later extended to many other placental mammals [23]. The Tur_tru_Illumina_hap_v1 assembly clearly illustrates and confirms the expected synteny.

Methods

Sample Collection and DNA Extraction: The sample for this study came from a female Atlantic bottlenose dolphin (Sample ID 04329), captive born at SeaWorld of Orlando, Orlando, Florida from wild male and female Atlantic bottlenose dolphins. The animal was 36 years old at blood collection with a healthy medical history. Blood was collected using Qiagen- PAXgene™ Blood DNA Tubes (Qiagen). High molecular weight genomic DNA was isolated using the Illumina- MasterPure™ DNA Purification Kit and subsequently quantified and qualified using Quant-iT™ dsDNA Kit and E-Gel™ EX Agarose Gel (ThermoFisher).

Collecting Sequence Data

Paired End (PE) libraries. We generated the 450bp and 800bp PE libraries using the Illumina TruSeq® PCR-free DNA Sample Prep kit. The protocol was slightly modified at fragmentation and double-size selection steps by adjusting the Covaris DNA shearing protocols and by empirically titrating the ratios of SPRI magnetic beads over

DNA to obtain insert sizes around 450bp and 800bp. We then evaluated the libraries for insert size and yield using Agilent Bioanalyzer and real-time qPCR assay, using Illumina DNA Standards and primer master mix qPCR kit (KAPA Biosystems, Roche), then normalized to 2nM prior to clustering and sequencing. Both the 450bp and 800 bp libraries were then denatured and diluted to 8pM and 12pM respectively. The 800bp PE library was clustered and sequenced on the HiSeq 2000, using the Illumina HiSeq Cluster and SBS v4 kits for PE 2x160bp reads. The 450bp PE library was clustered and sequenced on the HiSeq 2500 v2 Rapid Run mode using the HiSeq Rapid Cluster and SBS v2 kits for PE 2x250bp reads.

Mate Pair (MP) libraries. To maximize sequence diversity and genome coverage, three separate MP libraries were constructed corresponding to 2-5Kb, 5-7Kb and 7-10Kb insert sizes using the Nextera® MP Library Preparation Kit according to the manufacturer's instructions (Illumina). All three libraries were generated from a single input of 4ug of genomic DNA size-selected on a 0.8% E-gel (Invitrogen). Proper sizing of gel-extracted products was confirmed using the Bioanalyzer High Sensitivity chip (Agilent) and 600ng was subsequently used as input for circularization. Following library preparation, the Bioanalyzer was used to confirm library quality. Each of the three libraries were quantified by qPCR (KAPA Biosystems Library Quantification Kit, Roche), denatured and diluted to 200pM after size-adjustment according to Bioanalyzer results, and clustered on the cBot (Illumina) according to the manufacturer's instructions. 2x150bp of Illumina paired-end sequencing was performed on the HiSeq 4000 using the HiSeq 3000/4000 Cluster and SBS kits.

10x Chromium library. Genomic DNA quality was assessed by pulsed-field gel electrophoresis to determine suitability for 10X Chromium library preparation (10X Genomics). 1.125ng of input was used for library preparation according to the manufacturer's instructions without size-selection. Final library concentration was determined by qPCR (KAPA Library Quantification Kit, Roche) and size-adjusted according to Bioanalyzer DNA 100 chip (Agilent) results. 2x150bp of Illumina paired-end sequencing with an 8-base index read was performed on the HiSeq 4000 using the HiSeq 3000/4000 Cluster and SBS kits.

Genome Assembly

Genome assembly was conducted using the DeNovoMAGIC^{TM} software platform (NRGene, Nes Ziona, Israel). This is a proprietary DeBruijn-graph-based assembler that was used to produce assemblies of several challenging plant genomes such as corn [1] and ancestral wheat Aegilops tauschii [3]. The following outlines design of the assembler, and steps of the assembly process.

Reads pre-processing. In the pre-processing step we first removed PCR duplicate reads, and trimmed Illumina adaptor AGATCGGAAGAGC and Nextera® linker (for MP library) sequences. We then merged the PE 450bp 2×250bp overlapping reads with minimal required overlap of 10bp to create stitched reads (SRs) using the approach similar to the one implemented in the Flash software [27].

Error correction. We scanned through all merged reads to detect and filter out reads with apparent sequencing errors by examining k-mers (k=24) in the reads and looking for low abundance k-mers. We have high coverage data (~450x), with each read yielding 127 (150-24+1) to 227 (250-24+1) K-mers. Thus average 24-mer coverage is at least 300x. 24-mers that only appear less than 10 times in the set of reads likely contain errors. We did not use the reads that contain these low abundance k-mers for building initial contigs.

Contig assembly. The first step of the assembly consists of building a De Bruijn graph (kmer=127 bp) of contigs from all filtered reads. Next, paired end and MP reads are used to find reliable paths in the graph between contigs for repeat resolving and contigs extension. 10x barcoded reads were mapped to

contigs to ensure that adjacent contigs were connected only when there is evidence that those contigs originate from a single stretch of genomic sequence (reads from the same two or more barcodes were mapped to the same contigs).

Split phased/un-phased assembly processes. Two parallel assemblies take place to complete the phased and un-phased assembly result. The phased assembly process utilizes the complete set of contigs. In the un-phased assembly process, the homologous contigs are identified and one of the homologs is filtered out, leaving a subset of the homozygous and one of the homologous contigs in heterozygous regions. The linking information of both homologous contigs is kept through the assembly process of the un-phased assembly, usually enabling longer un-phased scaffolds.

Scaffolding. All the following steps are done in parallel for both the phased and un-phased assemblies. Contigs were linked into scaffolds with PE and MP information, estimating gaps between the contigs according to the distance of PE and MP links. In addition, for the phased assembly, 10x data were used to validate and support correct phasing during scaffolding.

Gap filling. A final gap fill step used PE and MP links and De Bruijn graph information to locally construct a unique path through the graph connecting the gap edges. The path was used to close the gap if it was unique and its length was consistent with the gap size estimate.

Scaffold split/merge. We used 10x barcoded reads to refine and merge scaffolds. All barcoded 10x reads were mapped to the assembled scaffolds. Clusters of reads with the same barcode mapped to adjacent contigs in the scaffolds were identified to be part of a single long molecule. Next, each scaffold was scanned with a 20kb length window to ensure that the number of distinct clusters that cover the entire window (indicating a support for this 20kb connection by several long molecules) is statistically significant with respect to the number of clusters that span the left and the right edge of the window. If there was a statistically significant disagreement in the coverage by the clusters over the window, we broke the scaffold at the two edges of the window. Finally, the barcodes that were mapped to the scaffold edges (first and last 20kb sequences) were compared to generate a graph of scaffolds. The scaffolds are nodes and the edges are links connecting nodes with more than two common barcodes on the ends. We broke the links to the nodes that had more than two links and output the resulting linear paths in the scaffold graph as final scaffolds.

Summary

We show that Tur_tru_Illumina_hap_v1 is more complete and accurate compared to the current best reference Tur_tru v1, based on the amount and composition of sequence, the consistency of the MP alignments to the assembled scaffolds, and on the analysis of conserved single-copy mammalian orthologs. The additional 12.5% of sequence data identified and assembled here was found to contain 165 additional BUSCO alignments as compared to the latest published assembly Tur_tru v1. The large scaffolds represented by Tur_tru_Illumina_hap_v1 enabled and confirmed expected synteny to human chromsome 1. The phased de novo assembly Tur_tru_Illumina_phased_v1 is of the first publicly available and it provides the community with novel ways to explore the heterozygous nature of the dolphin genome. These findings illustrate the impact of improved sample preparation and improved de novo assembly methods on progress toward more complete and accurate reference quality genomes. Better quality assemblies will improve our understanding of gene structure, function and evolution in mammalian species.

Table 1. Summary of the sequencing data collected to create Tur_tru_Illumina_hap_v1 and Tur_tru_Illumina_phased_v1.

Library type	Read Length	Insert Size	Genomic Coverage
**PCR-free**	2x250bp	450bp	101x
**PCR-free**	2x160bp	800bp	123x
**Mate-Pair**	2x150bp	2-4Kbp (peak 4.2Kbp)	37x
**Mate-Pair**	2x150bp	5-7Kbp (peak 6.0Kbp)	61x
**Mate-Pair**	2x150bp	8-10Kbp (peak 9.9Kbp)	58x
**10X Chromium**	2x150bp	-	70x

Table 2. Comparison of quantitative statistics for different assemblies of the bottlenose dolphin. The total sequence listed excludes Ns (ambiguous nucleotides). Ns were also squeezed out from the scaffolds for N50 computations. We used genome size of 2,383,130,043bp equal to the total amount of sequence in the scaffolds of the bigger haploid assembly, for comparison of the N50 contig and scaffold sizes between the two assemblies. The Tur_tru_Illumina_hap_v1 and Tur_tru v1 assemblies have comparable scaffold N50 sizes, and Tur_tru v1 has bigger contigs. The Tur_tru_Illumina_hap_v1 assembly has more sequence and our BUSCO analysis (Table 3) shows that it is likely more complete. The N50 comparisons to the haplotype-resolved Tur_tru_Illumina_phased_v1 assembly are shown for completeness, computed with 2x genome size (2*2,383,130,043 = 4,766,260,086bp).

	Tur_tru v1	Tur_tru_Illumina_hap_v1	Tur_tru_Illumina_phased_v1
**Total sequence**	2,120,283,832	2,383,130,043	4,678,362,582
**# of scaffolds**	2647	481	98,209
**Longest scaffold**	96,299,184	83,924,496	10,429,594
**Scaffold N50**	23,564,561	26,997,441	777,432
**Scaffold L50**	26	30	1,509
**# of contigs**	116,650	139,544	355,974
**Longest contig**	403,070	320,783	298,006
**Contig N50**	37,749	30,985	25,997
**Contig L50**	17,321	23,199	53,738
**GC content**	40.85	41.25	41.95

Table 3. Comparison of BUSCO 3.0.2 Mammalia single copy orthologs among the three Dolphin assemblies. The table shows that the Tur_tru_Illumina_hap_v1 assembly is more complete, with 110 fewer missing single-copy orthologs compared to the Tru_tru v1 assembly. The Tur_tru_Illumina_hap_v1 assembly has 43 extra duplicated orthologs, which possibly points to incomplete filtering of redundant haplotypes. While Tur_tru v1 assembly has bigger contigs, the Tur_tru_Illumina_hap_v1 assembly has many fewer fragmented BUSCOs. The haplotype-resolved Tur_tru_Illumina_phased_v1 assembly is less contiguous and less complete. As expected, more than half of the complete BUSCOs are duplicated, corresponding to the two resolved haplotypes.

BUSCOs	**Tur_tru v1**	**Tur_tru_Illumina_hap_v1**	**Tur_tru_Illumina_phased_v1**
Complete	3,647	3,837	3,537
Complete single-copy	3,614	3,760	1,310
Complete duplicated	33	77	2,227
Fragmented	187	107	301
Missing	270	160	266
Total	4,104	4,104	4,104

Table 4. Comparison of the number of mate pairs (MPs) from 5-7Kbp library uniquely aligned to Tur_tru_Illumina_hap_v1, Tur_tru_Illumina_phased_v1 and Tur_tru v1 assemblies. The alignments were done with Bowtie2. Only the reads that mapped **uniquely** were used for this computation, thus the number of MPs uniquely mapping to haplotype resolved assembly is much smaller. Same scaffold means that both mates mapped to the same scaffold; happy mates aligned in the correct orientation with mate distance within 5 standard deviations from the mean; misoriented mates aligned in wrong orientation; long mates aligned with the distance between the mates exceeding 5 standard deviations; short mates aligned with the distance of less than 1000bp. Same scaffold mate ALL is the total number of all mate pairs where both mates aligned to the same scaffold.

	Tur_tru_Illumina_hap_v1	Tur_tru_Illumina_phased_v1	Tur_tru v1
**Same scaffold mate ALL**	228,285,253	73,514,546	219,277,963
**Same scaffold happy**	125,224,663	37,248,420	117,942,390
**Same scaffold misoriented**	158,547	51,533	1,164,118
**Same scaffold long**	82,948	9,183	274,458
**Same scaffold short**	102,819,095	36,205,410	99,896,997
**Mates aligned to different scaffolds**	5,629,393	7,438,471	5,576,802
**One mate in the pair aligned**	61,284,891	26,228,913	68,707,025

Availability of data

The dolphin assembly Tur_tru_Illumina_hap_v1 has been deposited at NCBI under BioProject PRJNA476133, accession QMGA00000000. The dolphin assembly Tur_tru_Illumina_phased_v1 has been deposited at NCBI under BioProject PRJNA478376, accession QUXD00000000. Both assemblies are also available on the public FTP site ftp://ftp.ccb.jhu.edu/pub/alekseyz/Tur_tru_Illumina_v1/.

Competing interest

KV and CTL were both full-time employees of Illumina at the time this work was completed. Illumina is the company responsible for low cost high accuracy DNA sequencing. GBZ, KB, and TB are employees of NRGene, a company providing software analysis tools for de novo assembly.

Author contributions

KVM, CTL and MMV designed the project. KVM, CTL and AZ wrote the manuscript. GBZ, TB and KB generated genome assemblies. AZ conducted validation, MP consistency analysis, human chromosome 1 and BUSCO analyses, and submitted the genomes to NCBI; JSL provided the blood sample; JL, AN, MR, MG, EJ, and BS processed samples, generated sequencing and completed quality checks on sequence data; and all authors contributed to editing the manuscript.

Acknowledgement

This project was sponsored by Illumina, Inc. and NRGene. The authors acknowledge the support of the following people: Tristan Orpin and Matt Posard previously at Illumina, Inc.; Mark Van Oene, Feng Chen, Christine Ching, Shu Boles, Zheng Xu, Joey Flores, Kan Nobuta, Brad Sickler, Courtney McCormick, Christopher Haynes, Jennifer Bernet, and Nathalie Mouttham from Illumina, Inc.; Melissa Katigbak and Shara Fisler from Ocean Discovery Institute; Todd Schmitt from SeaWorld; Edwin Hauw, Deanna Church, and John Stuelpnagel from 10x Genomics.

Figure 1. Venn diagram of BUSCOs present in two dolphin assemblies. Out of 4,104 BUSCOs in mammalia set, 105 are missing from both assemblies. Our assembly has 165 BUSCOs not present in Tur_tru v1 and Tur_tru v1 has 55 BUSCOs that are not present in our assembly.

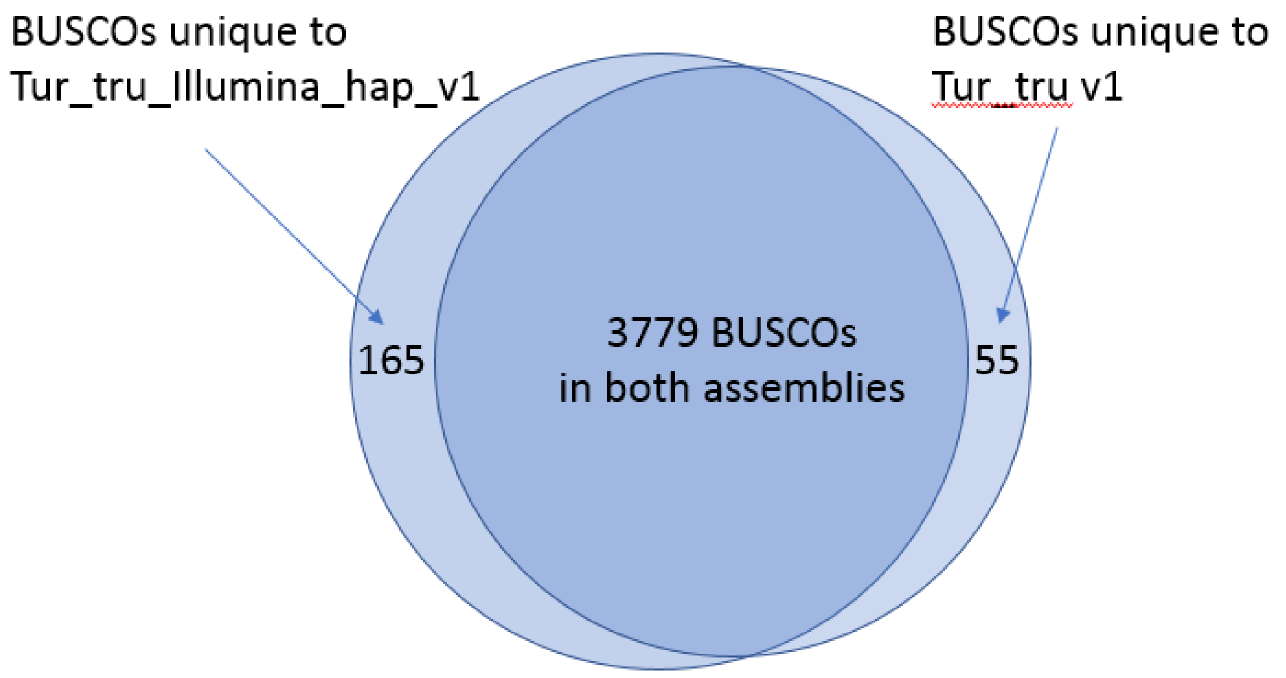

Figure 2. An example mummerplot of the alignments of the phased assembly to the "haploid" one, spanning about 15Mbp of sequence of scaffold314. The circles represent contig ends with lines joining them representing aligned sequence. The color indicates direction of the alignment with red and blue forward and reverse respectively. We show that for most locations on the x-axis (haploid assembly coordinate) there are two alignments on the y-axis corresponding to the two phased haplotypes. The small number of regions with a single contig aligning represent long homozygous regions of the genome that we were unable to phase.

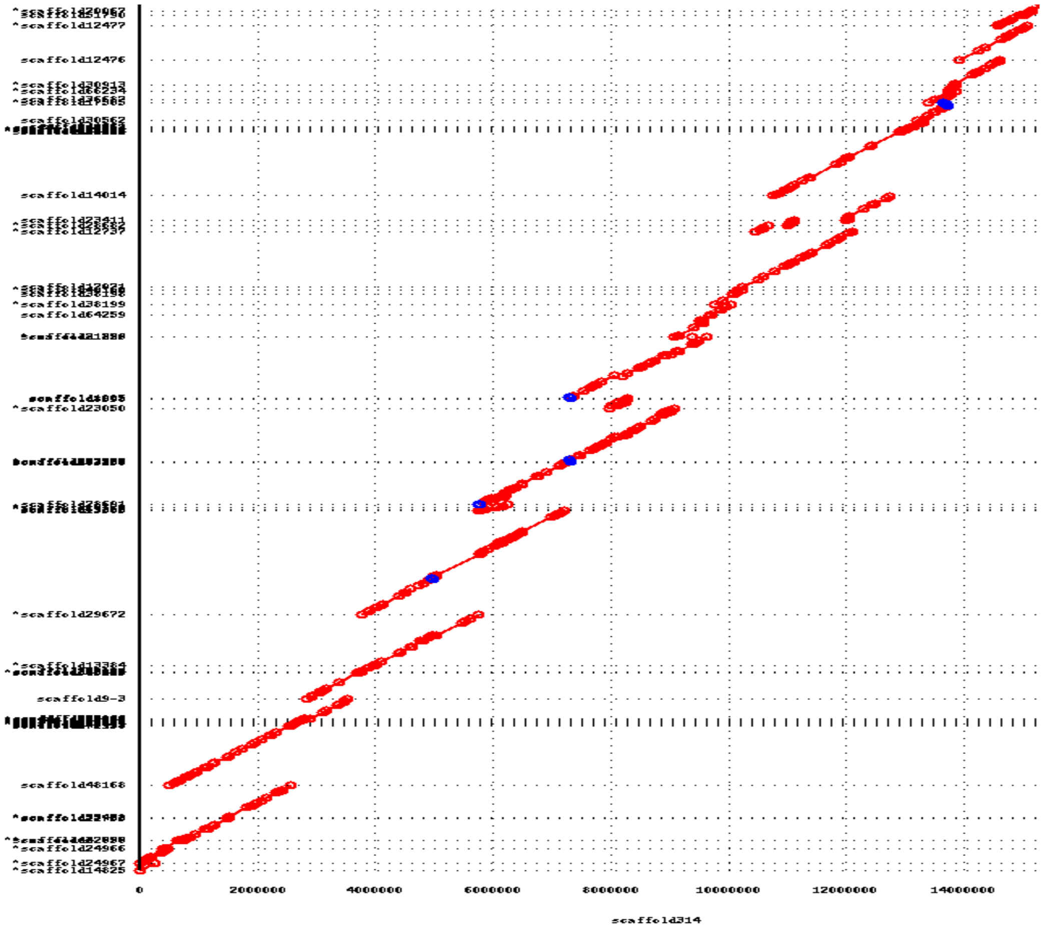

Figure 3. This figure shows the alignment of the Tur_tru_Illumina_hap_v1 assembly to the human GRCH38 reference (primary chromosomes only). Each dot represents an alignment with red indicating forward direction and blue indicating reverse direction. Human reference coordinates are on the X axis and Tur_tru_Illumina_hap_v1 assembly alignment coordinates are on the Y axis. Panel (a) shows alignment of the entire assembly to the human reference with alignments to human chromosome 1 highlighted by the black box. One can clearly see the synteny that is present between the dolphin scaffolds and human chromosome 1. No other human chromosome shows clear synteny. Dolphin scaffolds with syntenic alignments spanning over 50% of the scaffold were extracted. Alignments only to human chromosome 1 are presented in panel (b).

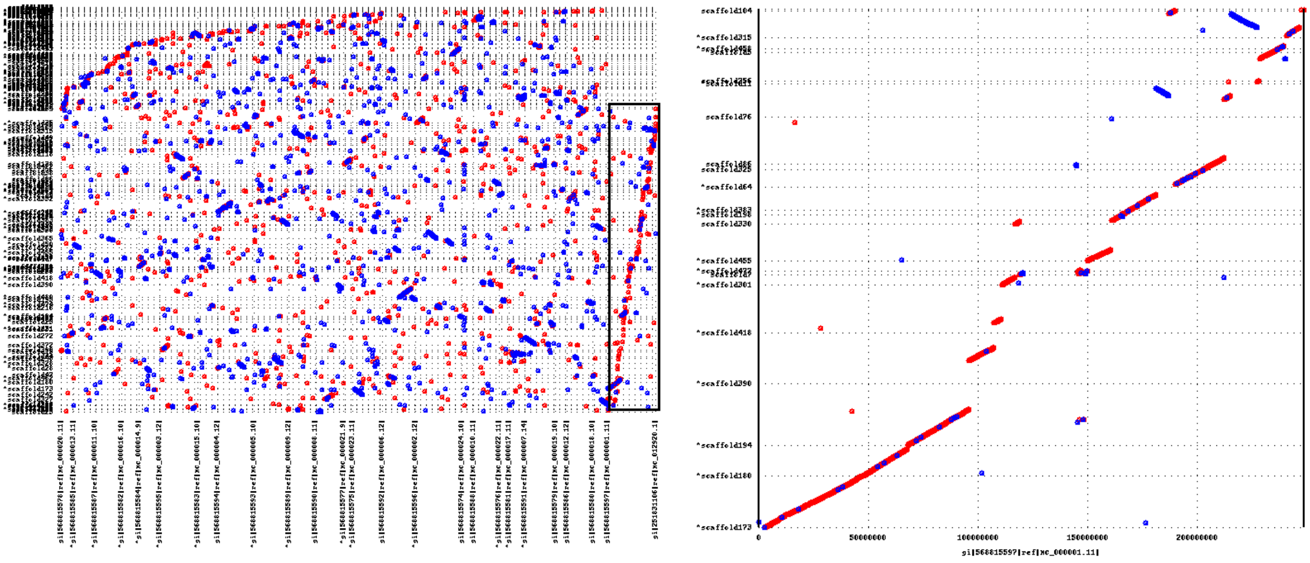

(a) (b)

References

1. Hirsch CN, Hirsch CD, Brohammer AB et al. Draft Assembly of Elite Inbred Line PH207 Provides Insights into Genomic and Transcriptome Diversity in Maize. Plant Cell 2016;28:2700-2714

2. Avni R, Nave M, Barad O et al. Wild emmer genome architecture and diversity elucidate wheat evolution and domestication. Science 2017;357:93-97

3. Luo MC, Gu YQ, Puiu D et al. Genome sequence of the progenitor of the wheat D genome Aegilops tauschii. Nature 2017;551:498-502

4. Zimin A, Stevens KA, Crepeau MW et al. An improved assembly of the loblolly pine mega-genome using long-read single-molecule sequencing. GIGAScience 2017;6:1-4

5. Genome 10K Community of Scientists. Genome 10K: A Proposal to Obtain Whole-Genome Sequence for 10000 Vertebrate Species. Journal of Heredity 2009;100(6):659-674

6. Koepfli K, Paten B, Antunes A et al. The Genome 10K Project: A Way Forward Further. Annual Review of Animal Biosciences 2015;3:57-111

7. Lewin HA, Robinson GE, Kress WJ et al. Earth BioGenome Project: Sequencing life for the future of life. Proceedings of the National Academy of Sciences 2018;115(17):4325-4333

8. Mohr DW, Naguib A, Weisenfeld N et al. Improved *de novo* Genome Assembly: Linked-Read Sequencing Combined with Optical Mapping Produce a High Quality Mammalian Genome at Relatively Low Cost. bioRxiv 2017;128348

9. Armstrong EE, Taylor RW, Prost S et al. Entering the era of conservation genomics: Cost-effective assembly of the African wild dog genome using linked long reads. bioRxiv 2017;195180

10. Hammond PS, Bearzi G, Bjørge A et al. *Tursiops truncatus*. The IUCN Red List of Threatened Species 2012:e.T22563A17347397. http://dx.doi.org/10.2305/IUCN.UK.2012.RLTS.T22563A17347397.en.

11. Rosel PE, Hancock-Hanser BL, Archer FI et al. Examining metrics and magnitudes of molecular genetic differentiation used to delimit cetacean subspecies based on mitochondrial DNA control region sequence. Special Issue: Delimiting subspecies using primarily genetic data. Marine Mammal Science 2017;33(S1):76-100

12. McGowen MR, Grossman LI, Wildman DE. Dolphin genome provides evidence for adaptive evolution of nervous system genes and a molecular rate slowdown. Proc. R. Soc. B 2012;279:3643–3651

13. Venn-Watson S, Carlin K, Ridgway S. Dolphins as animal models for type 2 diabetes: sustained, post-prandial hyperglycemia and hyperinsulinemia. Gen Comp Endocrinol 2011; 170(1):193-9

14. Venn-Watson S, Smith CR, Stevenson S et al. Blood-Based Indicators of Insulin Resistance and Metabolic Syndrome in Bottlenose Dolphins (*Tursiops truncatus*). Front Endocrinol (Lausanne) 2013;4:136

15. Venn-Watson S. Dolphins as animal models for type 2 diabetes: sustained, post-prandial hyperglycemia and hyperinsulineia. Frontiers in Endocrinology 2014; 227

16. Neely BA, Debra L. Ellisor DL et al. Proteomics as a metrological tool to evaluate genome annotation accuracy following de novo genome assembly: a case study using the Atlantic bottlenose dolphin (*Tursiops truncatus*). bioRxiv 2018;254250

17. Sobolesky P, Parry C, Boxall B et al. Proteomic analysis of non-depleted serum proteins from bottlenose dolphins uncovers a high vanin-1 phenotype. Scientific reports 2016;26(6):33879

18. Venn-Watson S, Smith CR, Gomez F et al. Physiology of aging among healthy, older bottlenose dolphins (*Tursiops truncatus*): comparisons with aging humans. J Comp Physiol B. 2011;181(5):667-80

19. Lindblad-Toh K, Garber M , Zuk O et al. A high-resolution map of human evolutionary constraint using 29 mammals. Nature 2011;478:10530

20. Foote AD, Liu Y, Thomas GWC et al. Convergent evolution of the genomes of marine mammals.2014. Nature Genetics 2014;47,3: 272-275

21. Marks P, Garcia S, Alvaro Martinez A et al. Resolving the Full Spectrum of Human Genome Variation using Linked-Reads. bioRxiv 2018;230946

22. Bielec PE, Gallagher DS, Womack JE et al. Homologies between human and dolphin chromosomes detected by heterologous chromosome painting. Cytogenetic and Genome Research 1998;81(1):18-25

23. Murphy WJ, Frönicke L, O'Brien SJ et al. The origin of human chromosome 1 and its homologs in placental mammals. Genome research. 2003;13(8):1880-8

24. Marçais G, Delcher AL, Phillippy AM et al. MUMmer4: A fast and versatile genome alignment system. PLOS 2018;1005944

25. Putnam NH, O'Connell BL, Stites JC et al. Chromosome-scale shotgun assembly using an in vitro method for long-range linkage. Genome Research 2016;26(3):342-50

26. Schneider VA, Graves-Lindsay T, Howe K et al. Evaluation of GRCh38 and de novo haploid genome assemblies demonstrates the enduring quality of the reference assembly. Genome research 2017;27(5):849-64

27. Magoč T, Salzberg SL. FLASH: fast length adjustment of short reads to improve genome assemblies. Bioinformatics 2011;27(21):2957-63

28. Langmead B, Salzberg SL. Fast gapped-read alignment with Bowtie 2. Nature methods. 2012;9(4):357-359. doi:10.1038/nmeth.1923.

 Click here to access/download;Figure;figure1.PNG

Figure 1. Venn diagram of BUSCOs present in two dolphin assemblies. Out of 4,104 BUSCOs in mammalia set, 105 are missing from both assemblies. Our assembly has 165 BUSCOs not present in Tur_tru v1 and Tur_tru v1 has 55 BUSCOs that are not present in our assembly.

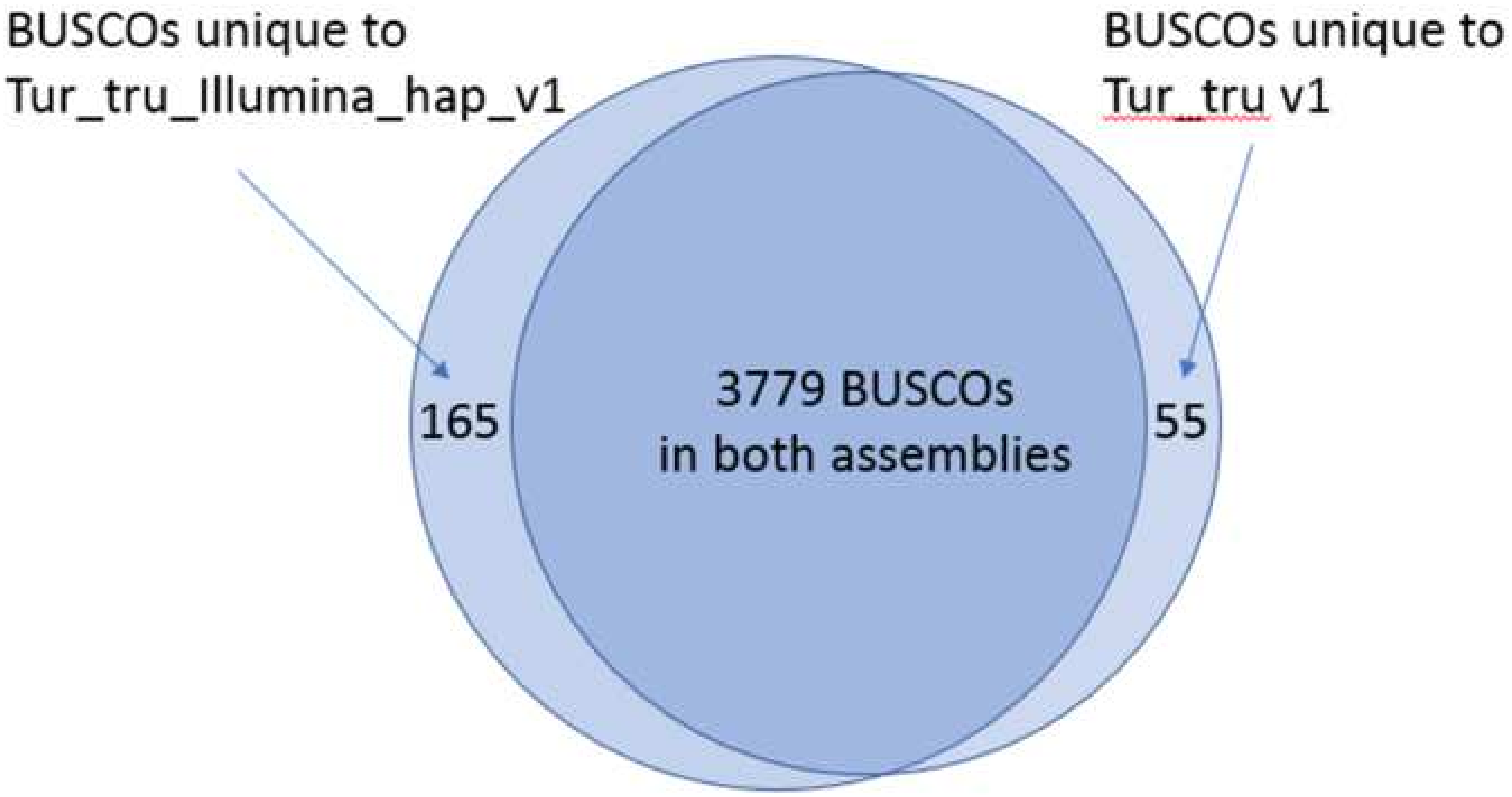

Click here to access/download;Figure;figure2.PNG

Figure 2. An example mummerplot of the alignments of the phased assembly to the "haploid" one, spanning about 15Mbp of sequence of scaffold314. The circles represent contig ends with lines joining them representing aligned sequence. The color indicates direction of the alignment with red and blue forward and reverse respectively. We show that for most locations on the x-axis (haploid assembly coordinate) there are two alignments on the y-axis corresponding to the two phased haplotypes. The small number of regions with a single contig aligning represent long homozygous regions of the genome that we were unable to phase.

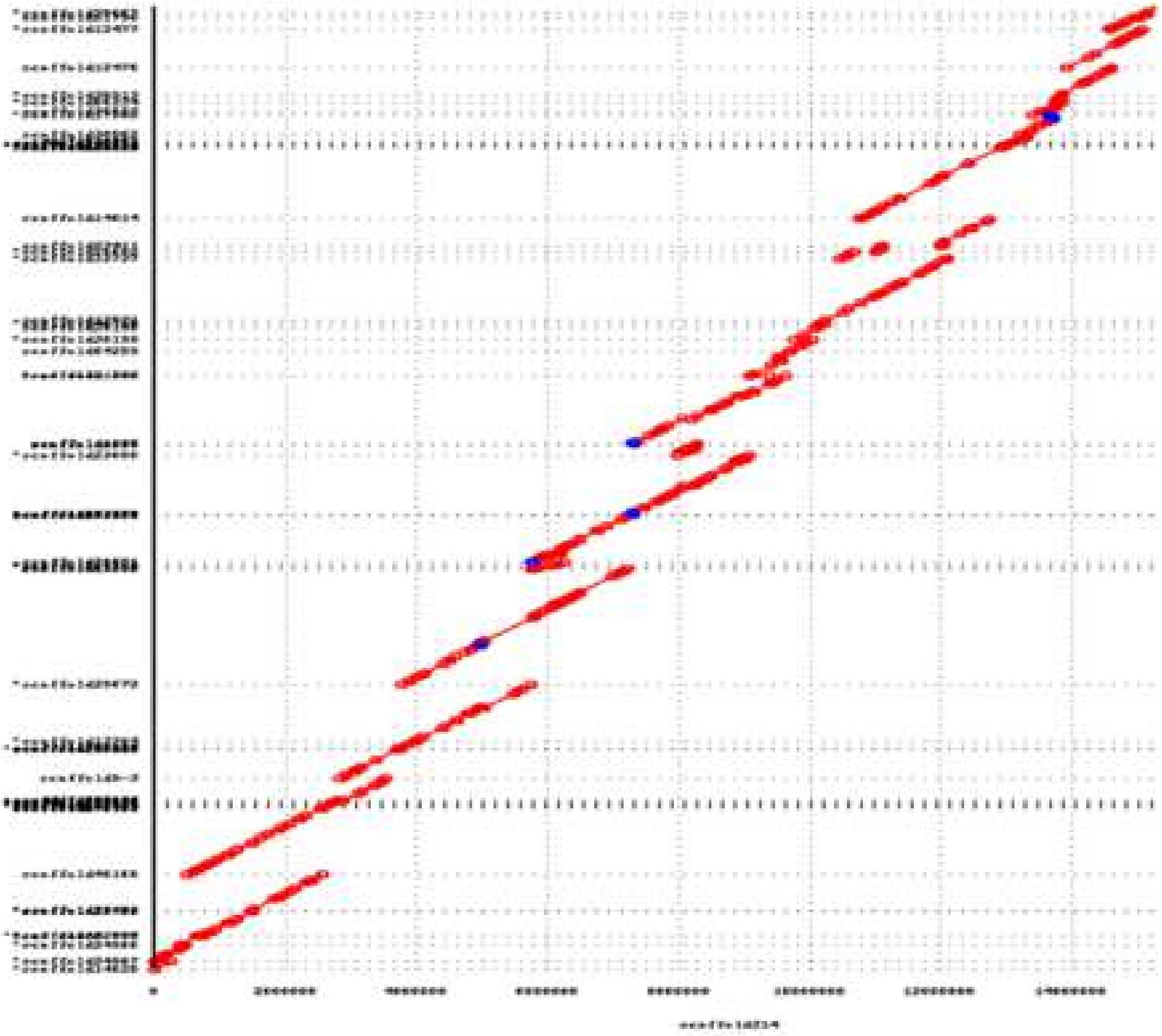

Click here to access/download;Figure;figure3.PNG

Figure 3. This figure shows the alignment of the Tur_tru_Illumina_hap_v1 assembly to the human GRCH38 reference (primary chromosomes only). Each dot represents an alignment with red indicating forward direction and blue indicating reverse direction. Human reference coordinates are on the X axis and Tur_tru_Illumina_hap_v1 assembly alignment coordinates are on the Y axis. Panel (a) shows alignment of the entire assembly to the human reference with alignments to human chromosome 1 highlighted by the black box. One can clearly see the synteny that is present between the dolphin scaffolds and human chromosome 1. No other human chromosome shows clear synteny. Dolphin scaffolds with syntenic alignments spanning over 50% of the scaffold were extracted. Alignments only to human chromosome 1 are presented in panel (b).

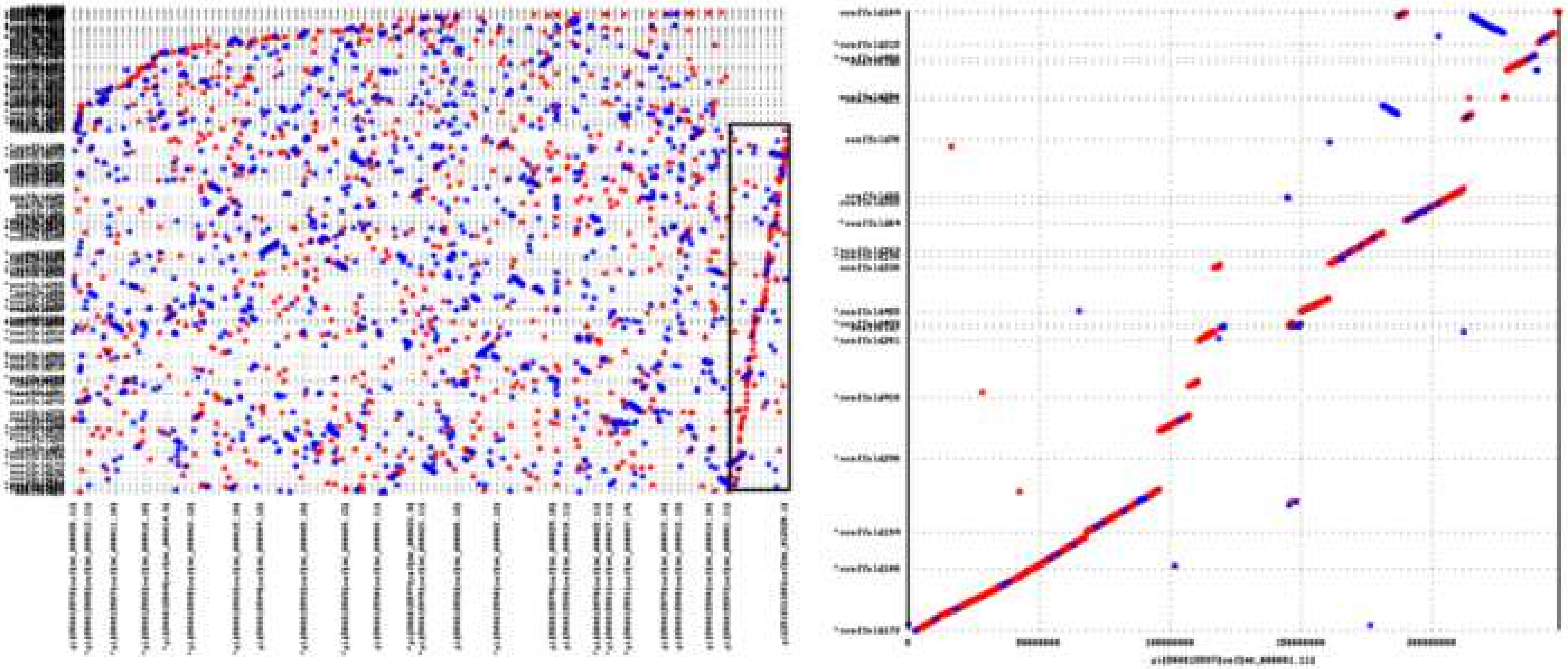

(a) (b)

 Click here to access/download;Personal Cover;Response to Reviews_v2_accepted.docx

We thank the reviewers for their careful review of the paper and constructive comments. We feel that addressing the comments has made the manuscript significantly stronger. We updated the tables and added two additional figures to the manuscript. Below we provide point-by-point responses to the reviewers' reports and list changes to the text that we have made in response to the comments. We have made changes to the paper using the "Track changes" feature in Word and we submit the revised version with changes highlighted. We also listed major changes in the responses.

Reviewer #1: "New de novo assembly of the Atlantic bottlenose dolphin (Tursiops truncatus) improves genome completeness and provides haplotype phasing" reports on improved haploid and diploid assemblies for the dolphin. The two major claims are: "Tur_tru_Illumina_hap_v1 is more complete and accurate compared to the current best reference based on the amount and composition of sequence, the consistency of the mate pair alignments to the assembled scaffolds, and on the analysis of conserved single-copy mammalian orthologs", and "Tur_tru_Illumina_dip_v1 is of the highest quality available for this species and provides the community with novel and accurate ways to explore the heterozygous nature of the dolphin genome".
This paper does not compare different sequencing technologies for sequencing the same genome such as 10X versus Oxford Nanopore. Nor does it compare different assemblers using the same data. This is a simple straight forward paper reporting an improved haplotype assembly and the first partially resolved diploid assembly for T. truncates. The three key sections are: Genome assembly comparison, Assembly validation through MP consistency, and Haplotype resolution. In the Genome assembly comparison section, the best existing assembly is aligned to the haplotype assembly using the MUMmer4 package which shows that there is much more unique sequence in the new assembly while maintaining similar contiguity statistics. BUSCO analysis shows that this unique additional sequence includes valuable protein coding gene regions. The new assembly is not missing any BUSCO genes from the existing assembly. The conclusion that the haplotype assembly is an improvement seems valid although the authors note there is some failure to collapse haplotypes 34 additional BUSCO genes are duplicated in the new "haplotype" assembly. Some BUSCO analysis of the diploid genome is also presented which is not as encouraging with 393 missing BUSCOs and only 2079 of the 3371 found BUSCOs duplicated showing that only partial haplotype resolution is achieved. In the Assembly validation through MP consistency section, the MP analysis shows that the new assembly is better than the existing assembly and plausible reasons for that are hypothesized.

1. The authors should however include a caveat that since the species level structural and transposon insertional variation is not known and since different individuals were assembled that some of the better MP statistics for the new assembly could be due to the use of read pairs from the individual sequenced for the new assembly.

   Response:
   Starting Line 214, We revised the text to: … Tur_tru v1 compared to Tur_tru_Illumina_hap_v1 (Table 4). Of course, some level of discrepancy is expected, because the two assemblies represent two different individuals with unknown level of structural variation between them. However, in concert, the two different categories may also suggest a possibility of a relatively higher number of locally mis-ordered or misoriented contigs in the scaffolds of Tur_tru v1 assembly. …

2. A major problem however is that table 4 does not support the text or at least is not explained in the text. Some
category of mate pair status must be being left out since there are many more mate pairs claimed in various categories for Tur_tru_v1 than for Tur_tru_Illumina_hap_v1. Based on the text one would expect more "Same scaffold happy" MPs for Tur_tru_Illumina_hap_v1 but this is not the case. Also the text claims Tur_tru_v1 has 8 times more "Same scaffold misoriented" MPs when it doesn't even have 7 times as many.

   Response:
   We agree with the reviewer that there are more mate pairs claimed in all categories for Tur_tru_v1. The difference can be explained by the peculiarities of the MUMmer software that we used to produce the alignments. By default, if there is a high-identity repeat region in the assembly, Nucmer will not find any seeds for the alignment, because all alignments are initiated by looking for clusters of exact matching seeds in the reference genome (genome we align to) with minimum length of 20. If the reference has two regions of length >=20 that are identical, no seeds will be found there. Thus, an assembly that has two or more copies of repeat will have no reads mapping to either copy, while the assembly that collapsed repeat to one copy will have reads mapping to the same copy. To validate this hypothesis, we re-did the alignments using Bowtie2 short read aligner to align the same data to both assemblies. Bowtie2 does not have the same deficiency as MUMmer in mapping reads to repeat regions. We only used alignments of reads that mapped uniquely to the assembly to avoid noise in the results. All numbers in Table 4 have been updated, and we added new category where we list the sum of all mate pairs aligned to the same scaffold. Now we see that the number of mates aligned to both assemblies is similar, with alignment patterns that support the reasoning in the text. We updated the text to replace MUMmer4 by Bowtie2. We also added the analysis of the mate pair alignments to the haplotype phased assembly.

   Starting Line 232 We revised the text to: The haplotype phased assembly is much more fragmented, resulting in a higher relative number of mate pairs mapping to different scaffolds. However, when looking at the "internal" mate pairs, i.e. where both mates map at least 10Kb away from the scaffold ends, we see remarkable consistency with less than 0.5% of the mates mapped to the wrong scaffold (see next section). Since for this analysis we only used mates mapping uniquely to the assembly, and there are two copies of the genome in the assembly, the total number of mapped mates is much lower.

3. Finally in the Haplotype resolution system, MPs were again used to evaluate the amount of haplotype switching between scaffolds asserted to be haplotype separated. No MPs were found to map to different haplotype separated scaffolds. The problem with this section is that there may be ascertainment bias in that only large separated scaffolds were used. The number of MPs mapped is very small compared to the numbers in table 4 and again there is no accounting given for what happened to most mate pairs which should be covered in a table 5. I would assume that smaller
scaffolds are much more likely to not be separated as well. The same mate pair library

was mapped to the entire haplotype assembly so why not the entire diploid assembly? Also there is no evaluation of comparing the diploid assembly to the haploid assembly. Large separated scaffolds were apparently mapped but not smaller ones?

Response:
In haplotype separation it is typically easier to phase short regions (an example is a single SNP that is different between the two haplotypes, or in insertion in one of the haplotypes). However, when phasing large regions one has to be careful not to "jump" to a different haplotype while extending haploid sequence. This is why we originally looked at the largest haplotype-phased scaffolds for evaluation of the efficacy of haplotype separation. We changed the way we measure the efficacy of the haplotype resolution; the new text and results are as follows.

Starting Line 273 We changed the text to: We analyzed the quality of the haplotype phasing as follows. In haplotype phasing it is easy to phase small regions. For example, a single isolated SNP with no haplotype differences within 100 bp in both directions, can be trivially phased into two 201bp (or longer) contigs different by one base in the middle. It gets more difficult for larger contigs/scaffolds, where one must make sure that the contig/scaffold represents single haplotype and not a "mosaic" of haplotypes, that is the SNPs and other bigger haplotype differences are correctly "phased". To do that we mapped the mate pairs from the 5-7Kb mate pair library to all phased scaffolds using Bowtie2 [28], and then examined the "internal" mate pairs where both reads in each pair mapped to the assembly, and one read mapped within 10Kb away from the ends of the scaffold. This would imply that the other mate must map to the same scaffold and not its haplotype, if haplotype phasing is done properly. If it does not, then it indicates an apparent mis-assembly or failure to phase haplotypes. By measuring the number of "properly" aligned internal mates, where both mates aligned to the same scaffold vs. "improper" internal mates where the mates aligned to different scaffolds, one can measure the efficacy of the haplotype phasing. There were 35,697,369 pairs where both mates mapped properly to the same scaffold, while only 169,244 mapped improperly, that is to two different scaffolds. The percentage of improperly mapping mate pairs is only 0.5%, indicating that haplotype resolution was done properly.

4. In table 2 it is not clear if total sequence for the scaffolds includes Ns in the gaps but it would appear to. There is no discussion of why the diploid assembly is more than twice as big as the haploid assembly given that based on BUSCO the haplotype resolution is far from complete. Assuming gaps are included in the sizes the explanation seems obvious that many scaffolds overlap with or are contained in other scaffolds with contigs interleaving.
   I cannot recommend accepting this article until the discrepancies in table 4 are explained and a comparison of the haploid and diploid assemblies is given to try to explain the amount of haplotype resolution and the scaffold size discrepancy.

Response:
The statistics for the phased assembly in Table 2 were computed incorrectly. Thank you for helping us identify this discrepancy. They were computed on the pre-release draft version of the assembly that had redundant sequences. We re-computed the statistics on the latest version of

the assembly that was generated after the initial writing of that section of the paper and updated Table 2. The latest version is available from our ftp site listed in the paper and has been resubmitted to NCBI with accession QUXD00000000. We clarified the caption in Table 2 by adding "The total sequence listed excludes Ns." For confirmation, we re-computed the numbers to make sure N's are excluded from all N50 and size computations, because N50 numbers should reflect sizes of actual sequences without (sometimes) arbitrarily estimated gaps. Also we updated the numbers for the haploid assembly since they changed slightly after filtering the assembly for contaminants, which is part of NCBI submission process. The filtering results were reported to us after we submitted the manuscript.

5. I also strongly recommend that the authors tone down any claims for superiority of this assembly since it is not that much better than the existing one - better but not perfect. For example, the diploid assembly is touted as the "highest quality available for this species" but then later in the paper it is claimed it is the only diploid assembly for the species which makes the previous claim trivially true but misleading.

   Response:
   We agree that our text sounded misleading and we thank the reviewer for bringing that to our attention. Scientific writing should be state the facts clearly. We have changed the "highest" to the "first" in the following sentence in the abstract, and in the summary, and it now reads:

   Starting Line 47 We changed the text to: The phased de novo assembly Tur_tru_Illumina_dip_v1 is the first publicly available for this species and provides the community with novel and accurate ways to explore the heterozygous nature of the dolphin genome.

Reviewer #2: This manuscript provide new genome assemblies for Atlantic bottlenose dolphin based on new sequencing data set. Here are my comments:

1) It appears that the new assemblies did not leverage the old but valuable sequencing data based on Sanger and 454 reads, which together give about ~6X coverage of data. Such data might benefit the contiguity of the assembly a lot. And this is probably already reflected on the contig N50 size, which is only 30Kb in the new assembly (even hundreds coverage of reads were used), shorter than the previous assemblies based on the 454/sanger reads (37Kb). The authors need to discuss on this.

Response:
The old 454 and Sanger data was from a different animal. Using it would likely fracture assembly even further because of the structural/haplotype differences between the animals. Typically for a good assembly data from only one individual must be used.

2) It is not very easy to get the detailed information on what software were used on scaffolding, gap filling, phasing and scaffold splitting/merging. Did the authors used a

home-made software, or publicly available tools? Where to access these tools? what parameters were used?

Response:
The software that was used for this assembly is DeNovoMagic, it is proprietary and it was developed by NRGene LTD. In the paper we outline the steps that were followed by the software to produce the assembly. The software is not open source and it is not available for general use. However, it has been used before to produce many genome assemblies, such as maize and wheat, published now in major journals.

3) The names of Tur_tru_Illumina_hap_v1 and Tur_tru_Illumina_dip_v1 could be somewhat misleading: hap=haploid whereas the dip=diploid. May be haploid versus phased-haplotypes.

Response:
We understand the confusion. We changed the name of the phased assembly to Tur_tru_Illumina_phased_v1

4) The synteny analysis appears show no new finding or improvement over the old assembly (which may be due to the fact the old ones are already chromosome-level assembly?). If there is new findings/improvements, the authors are encouraged to show them.

Response:
Neither the old nor the new assembly are chromosome-level. Both are scaffold level assemblies. We show the synteny to human to illustrate the finding mentioned in the earlier literature.

5) There could be more comparative analysis between the new and old assemblies. It appears they are based on different data set. For example, some Venn figures could to use to show the differences between them. LIke, 1000 genes, 900 are both complete in new and old assemblies, while new assemblies has 60 more complete genes and the old have 40 more complete genes but they are not overlapped. And some examples of the genes could be provided.

Response:
This is an excellent suggestion, and we thank the reviewer for bringing this clarifying idea to our attention. We added the Venn diagram with the analysis of BUSCO genes to the paper as Figure 1.

--
