# Supplementary material for: New de novo assembly of the Atlantic bottlenose dolphin (Tursiops truncatus) improves genome completeness and provides haplotype phasing
Source: Gigascience. 2019 Jan 29;8(3):giy168. doi: 10.1093/gigascience/giy168 (PMC6443575; doi:10.1093/gigascience/giy168)
Supplement: Response_to_Reviewer_Comments_Original_Submission.pdf [file giy168_response_to_reviewer_comments_original_submission.pdf]

Author's Response To Reviewer Comments

Close

We also included a Word copy of this document in the re-submission.

We thank the reviewers for their careful review of the paper and constructive comments. We feel that addressing the comments has made the manuscript significantly stronger. We updated the tables and added two additional figures to the manuscript. Below we provide point-by-point responses to the reviewers' reports and list changes to the text that we have made in response to the comments. We have made changes to the paper using the "Track changes" feature in Word and we submit the revised version with changes highlighted. We also listed major changes in the responses.

Reviewer #1: "New de novo assembly of the Atlantic bottlenose dolphin (Tursiops truncatus) improves genome completeness and provides haplotype phasing" reports on improved haploid and diploid assemblies for the dolphin. The two major claims are: "Tur_tru_Illumina_hap_v1 is more complete and accurate compared to the current best reference based on the amount and composition of sequence, the consistency of the mate pair alignments to the assembled scaffolds, and on the analysis of conserved single-copy mammalian orthologs", and "Tur_tru_Illumina_dip_v1 is of the highest quality available for this species and provides the community with novel and accurate ways to explore the heterozygous nature of the dolphin genome".
This paper does not compare different sequencing technologies for sequencing the same genome such as 10X versus Oxford Nanopore. Nor does it compare different assemblers using the same data. This is a simple straight forward paper reporting an improved haplotype assembly and the first partially resolved diploid assembly for T. truncates. The three key sections are: Genome assembly comparison, Assembly validation through MP consistency, and Haplotype resolution. In the Genome assembly comparison section, the best existing assembly is aligned to the haplotype assembly using the MUMmer4 package which shows that there is much more unique sequence in the new assembly while maintaining similar contiguity statistics. BUSCO analysis shows that this unique additional sequence includes valuable protein coding gene regions. The new assembly is not missing any BUSCO genes from the existing assembly. The conclusion that the haplotype assembly is an improvement seems valid although the
authors note there is some failure to collapse haplotypes 34 additional BUSCO genes are duplicated in the new "haplotype" assembly. Some BUSCO analysis of the diploid genome is also presented which is not as encouraging with 393 missing BUSCOs and only 2079 of the 3371 found BUSCOs duplicated showing that only partial haplotype resolution is achieved. In the Assembly validation through MP consistency section, the MP analysis shows that the new assembly is better than the existing assembly and plausible reasons for that are hypothesized.
1. The authors should however include a caveat that since the species level structural and transposon insertional variation is not known and since different individuals were assembled that some of the better MP statistics for the new assembly could be due to the use of read pairs from the individual sequenced for the new assembly.

Response:
Starting Line 214, We revised the text to: … Tur_tru v1 compared to Tur_tru_Illumina_hap_v1 (Table 4). Of course, some level of discrepancy is expected, because the two assemblies represent two different individuals with unknown level of structural variation between them. However, in concert, the two different categories may also suggest a possibility of a relatively higher number of locally mis-ordered or misoriented contigs in the scaffolds of Tur_tru v1 assembly. …

2. A major problem however is that table 4 does not support the text or at least is not explained in the text. Some
category of mate pair status must be being left out since there are many more mate pairs claimed in various categories for Tur_tru_v1 than for Tur_tru_Illumina_hap_v1. Based on the text one would expect more "Same scaffold happy" MPs for Tur_tru_Illumina_hap_v1 but this is not the case. Also the text claims Tur_tru_v1 has 8 times more "Same scaffold misoriented" MPs when it doesn't even have 7 times as many.

Response:
We agree with the reviewer that there are more mate pairs claimed in all categories for Tur_tru_v1. The difference can be explained by the peculiarities of the MUMmer software that we used to produce the alignments. By default, if there is a high-identity repeat region in the assembly, Nucmer will not find any seeds for the alignment, because all alignments are initiated by looking for clusters of exact matching seeds in the reference genome (genome we align to) with minimum length of 20. If the reference has two regions of length >=20 that are identical, no seeds will be found there. Thus, an assembly that has two or more copies of repeat will have no reads mapping to either copy, while the assembly that collapsed repeat to one copy will have reads mapping to the same copy. To validate this hypothesis, we re-did the alignments using Bowtie2 short read aligner to align the same data to both assemblies. Bowtie2 does not have the same deficiency as MUMmer in mapping reads to repeat regions. We only used alignments of reads that mapped uniquely to the assembly to avoid noise in the results. All numbers in Table 4 have been updated, and we added new category where we list the sum of all mate pairs aligned to the same scaffold. Now we see that the number of mates aligned to both assemblies is similar, with alignment patterns that support the reasoning in the text. We updated the text to replace MUMmer4 by Bowtie2. We also added the analysis of the mate pair alignments to the haplotype phased assembly.

Starting Line 232 We revised the text to: The haplotype phased assembly is much more fragmented, resulting in a higher relative number of mate pairs mapping to different scaffolds. However, when looking at the “internal” mate pairs, i.e. where both mates map at least 10Kb away from the scaffold ends, we see remarkable consistency with less than 0.5% of the mates mapped to the wrong scaffold (see next section). Since for this analysis we only used mates mapping uniquely to the assembly, and there are two copies of the genome in the assembly, the total number of mapped mates is much lower.

3. Finally in the Haplotype resolution system, MPs were again used to evaluate the amount of haplotype switching between scaffolds asserted to be haplotype separated. No MPs were found to map to different haplotype separated scaffolds. The problem with this section is that there

may be ascertainment bias in that only large separated scaffolds were used. The number of MPs mapped is very small compared to the numbers in table 4 and again there is no accounting given for what happened to most mate pairs which should be covered in a table 5. I would assume that smaller
scaffolds are much more likely to not be separated as well. The same mate pair library was mapped to the entire haplotype assembly so why not the entire diploid assembly? Also there is no evaluation of comparing the diploid assembly to the haploid assembly. Large separated scaffolds were apparently mapped but not smaller ones?
Response:
In haplotype separation it is typically easier to phase short regions (an example is a single SNP that is different between the two haplotypes, or in insertion in one of the haplotypes). However, when phasing large regions one has to be careful not to "jump" to a different haplotype while extending haploid sequence. This is why we originally looked at the largest haplotype-phased scaffolds for evaluation of the efficacy of haplotype separation. We changed the way we measure the efficacy of the haplotype resolution; the new text and results are as follows.
Starting Line 273 We changed the text to: We analyzed the quality of the haplotype phasing as follows. In haplotype phasing it is easy to phase small regions. For example, a single isolated SNP with no haplotype differences within 100 bp in both directions, can be trivially phased into two 201bp (or longer) contigs different by one base in the middle. It gets more difficult for larger contigs/scaffolds, where one must make sure that the contig/scaffold represents single haplotype and not a "mosaic" of haplotypes, that is the SNPs and other bigger haplotype differences are correctly "phased". To do that we mapped the mate pairs from the 5-7Kb mate pair library to all phased scaffolds using Bowtie2 [28], and then examined the "internal" mate pairs where both reads in each pair mapped to the assembly, and one read mapped within 10Kb away from the ends of the scaffold. This would imply that the other mate must map to the same scaffold and not its haplotype, if haplotype phasing is done properly. If it does not, then it indicates an apparent mis-assembly or failure to phase haplotypes. By measuring the number of "properly" aligned internal mates, where both mates aligned to the same scaffold vs. "improper" internal mates where the mates aligned to different scaffolds, one can measure the efficacy of the haplotype phasing. There were 35,697,369 pairs where both mates mapped properly to the same scaffold, while only 169,244 mapped improperly, that is to two different scaffolds. The percentage of improperly mapping mate pairs is only 0.5%, indicating that haplotype resolution was done properly.

4. In table 2 it is not clear if total sequence for the scaffolds includes Ns in the gaps but it would appear to. There is no discussion of why the diploid assembly is more than twice as big as the haploid assembly given that based on BUSCO the haplotype resolution is far from complete. Assuming gaps are included in the sizes the explanation seems obvious that many scaffolds overlap with or are contained in other scaffolds with contigs interleaving.
I cannot recommend accepting this article until the discrepancies in table 4 are explained and a comparison of the haploid and diploid assemblies is given to try to explain the amount of haplotype resolution and the scaffold size discrepancy.

Response:
The statistics for the phased assembly in Table 2 were computed incorrectly. Thank you for helping us identify this discrepancy. They were computed on the pre-release draft version of the

assembly that had redundant sequences. We re-computed the statistics on the latest version of the assembly that was generated after the initial writing of that section of the paper and updated Table 2. The latest version is available from our ftp site listed in the paper and has been resubmitted to NCBI with accession QUXD00000000. We clarified the caption in Table 2 by adding "The total sequence listed excludes Ns." For confirmation, we re-computed the numbers to make sure N's are excluded from all N50 and size computations, because N50 numbers should reflect sizes of actual sequences without (sometimes) arbitrarily estimated gaps. Also we updated the numbers for the haploid assembly since they changed slightly after filtering the assembly for contaminants, which is part of NCBI submission process. The filtering results were reported to us after we submitted the manuscript.

5. I also strongly recommend that the authors tone down any claims for superiority of this assembly since it is not that much better than the existing one - better but not perfect. For example, the diploid assembly is touted as the "highest quality available for this species" but then later in the paper it is claimed it is the only diploid assembly for the species which makes the previous claim trivially true but misleading.

Response:
We agree that our text sounded misleading and we thank the reviewer for bringing that to our attention. Scientific writing should be state the facts clearly. We have changed the "highest" to the "first" in the following sentence in the abstract, and in the summary, and it now reads:

Starting Line 47 We changed the text to: The phased de novo assembly Tur_tru_Illumina_dip_v1 is the first publicly available for this species and provides the community with novel and accurate ways to explore the heterozygous nature of the dolphin genome.

Reviewer #2: This manuscript provide new genome assemblies for Atlantic bottlenose dolphin based on new sequencing data set. Here are my comments:

1) It appears that the new assemblies did not leverage the old but valuable sequencing data based on Sanger and 454 reads, which together give about ~6X coverage of data. Such data might benefit the contiguity of the assembly a lot. And this is probably already reflected on the contig N50 size, which is only 30Kb in the new assembly (even hundreds coverage of reads were used), shorter than the previous assemblies based on the 454/sanger reads (37Kb). The authors need to discuss on this.

Response:
The old 454 and Sanger data was from a different animal. Using it would likely fracture assembly even further because of the structural/haplotype differences between the animals. Typically for a good assembly data from only one individual must be used.

2) It is not very easy to get the detailed information on what software were used on scaffolding, gap filling, phasing and scaffold splitting/merging. Did the authors used a home-made software, or publicly available tools? Where to access these tools? what parameters were used?

Response:
The software that was used for this assembly is DeNovoMagic, it is proprietary and it was developed by NRGene LTD. In the paper we outline the steps that were followed by the software to produce the assembly. The software is not open source and it is not available for general use. However, it has been used before to produce many genome assemblies, such as maize and wheat, published now in major journals.

3) The names of Tur_tru_Illumina_hap_v1 and Tur_tru_Illumina_dip_v1 could be somewhat misleading: hap=haploid whereas the dip=diploid. May be haploid versus phased-haplotypes.

Response:
We understand the confusion. We changed the name of the phased assembly to Tur_tru_Illumina_phased_v1

4) The synteny analysis appears show no new finding or improvement over the old assembly (which may be due to the fact the old ones are already chromosome-level assembly?). If there is new findings/improvements, the authors are encouraged to show them.

Response:
Neither the old nor the new assembly are chromosome-level. Both are scaffold level assemblies. We show the synteny to human to illustrate the finding mentioned in the earlier literature.

5) There could be more comparative analysis between the new and old assemblies. It appears they are based on different data set. For example, some Venn figures could to use to show the differences between them. LIke, 1000 genes, 900 are both complete in new and old assemblies, while new assemblies has 60 more complete genes and the old have 40 more complete genes but they are not overlapped. And some examples of the genes could be provided.

Response:
This is an excellent suggestion, and we thank the reviewer for bringing this clarifying idea to our attention. We added the Venn diagram with the analysis of BUSCO genes to the paper as Figure 1.

--

Close
