# Supplementary material for: New de novo assembly of the Atlantic bottlenose dolphin (Tursiops truncatus) improves genome completeness and provides haplotype phasing
Source: Gigascience. 2019 Jan 29;8(3):giy168. doi: 10.1093/gigascience/giy168 (PMC6443575; doi:10.1093/gigascience/giy168)
Supplement: Reviewer_1_Report_Original_Submission -- Granger Gideon Sutton, Ph.D. — 8/17/2018 Reviewed [file giy168_reviewer_1_report_original_submission.pdf]

Reviewer Report

Title: New de novo assembly of the Atlantic bottlenose dolphin (Tursiops truncatus) improves genome completeness and provides haplotype phasing

Version: Original Submission **Date:** 8/17/2018

Reviewer name: Granger Gideon Sutton, Ph.D.

Reviewer Comments to Author:

"New de novo assembly of the Atlantic bottlenose dolphin (Tursiops truncatus) improves genome completeness and provides haplotype phasing" reports on improved haploid and diploid assemblies for the dolphin. The two major claims are: "Tur_tru_Illumina_hap_v1 is more complete and accurate compared to the current best reference based on the amount and composition of sequence, the consistency of the mate pair alignments to the assembled scaffolds, and on the analysis of conserved single-copy mammalian orthologs", and "Tur_tru_Illumina_dip_v1 is of the highest quality available for this species and provides the community with novel and accurate ways to explore the heterozygous nature of the dolphin genome".
This paper does not compare different sequencing technologies for sequencing the same genome such as 10X versus Oxford Nanopore. Nor does it compare different assemblers using the same data. This is a simple straight forward paper reporting an improved haplotype assembly and the first partially resolved diploid assembly for T. truncates. The three key sections are: Genome assembly comparison, Assembly validation through MP consistency, and Haplotype resolution. In the Genome assembly comparison section, the best existing assembly is aligned to the haplotype assembly using the MUMmer4 package which shows that there is much more unique sequence in the new assembly while maintaining similar contiguity statistics. BUSCO analysis shows that this unique additional sequence includes valuable protein coding gene regions. The new assembly is not missing any BUSCO genes from the existing assembly. The conclusion that the haplotype assembly is an improvement seems valid although the authors note there is some failure to collapse haplotypes 34 additional BUSCO genes are duplicated in the new "haplotype" assembly. Some BUSCO analysis of the diploid genome is also presented which is not as encouraging with 393 missing BUSCOs and only 2079 of the 3371 found BUSCOs duplicated showing that only partial haplotype resolution is achieved. In the Assembly validation through MP consistency section, the MP analysis shows that the new assembly is better than the existing assembly and plausible reasons for that are hypothesized. The authors should however include a caveat that since the species level structural and transposon insertional variation is not known and since different individuals were assembled that some of the better MP statistics for the new assembly could be due to the use of read pairs from the individual sequenced for the new assembly. A major problem however is that table 4 does not support the text or at least is not explained in the text. Some category of mate pair status must be being left out since there are many more mate pairs claimed in various categories for Tur_tru_v1 than for Tur_tru_Illumina_hap_v1. Based on the text one would expect more "Same scaffold happy" MPs for Tur_tru_Illumina_hap_v1 but this is not the case. Also the text claims Tur_tru_v1 has 8 times more "Same scaffold misoriented" MPs when it doesn't even have 7 times as many. Finally in the

Haplotype resolution system, MPs were again used to evaluate the amount of haplotype switching between scaffolds asserted to be haplotype separated. No MPs were found to map to different haplotype separated scaffolds. The problem with this section is that there may be ascertainment bias in that only large separated scaffolds were used. The number of MPs mapped is very small compared to the numbers in table 4 and again there is no accounting given for what happened to most mate pairs which should be covered in a table 5. I would assume that smaller scaffolds are much more likely to not be separated as well. The same mate pair library was mapped to the entire haplotype assembly so why not the entire diploid assembly? Also there is no evaluation of comparing the diploid assembly to the haploid assembly. Large separated scaffolds were apparently mapped but not smaller ones? In table 2 it is not clear if total sequence for the scaffolds includes Ns in the gaps but it would appear to. There is no discussion of why the diploid assembly is more than twice as big as the haploid assembly given that based on BUSCO the haplotype resolution is far from complete. Assuming gaps are included in the sizes the explanation seems obvious that many scaffolds overlap with or are contained in other scaffolds with contigs interleaving.
I cannot recommend accepting this article until the discrepancies in table 4 are explained and a comparison of the haploid and diploid assemblies is given to try to explain the amount of haplotype resolution and the scaffold size discrepancy.
I also strongly recommend that the authors tone down any claims for superiority of this assembly since it is not that much better than the existing one - better but not perfect. For example, the diploid assembly is touted as the "highest quality available for this species" but then later in the paper it is claimed it is the only diploid assembly for the species which makes the previous claim trivially true but misleading.

Level of Interest

Please indicate how interesting you found the manuscript: Choose an item.

Quality of Written English

Please indicate the quality of language in the manuscript: Choose an item.

Declaration of Competing Interests

Please complete a declaration of competing interests, considering the following questions:

- Have you in the past five years received reimbursements, fees, funding, or salary from an organisation that may in any way gain or lose financially from the publication of this manuscript, either now or in the future?
- Do you hold any stocks or shares in an organisation that may in any way gain or lose financially from the publication of this manuscript, either now or in the future?
- Do you hold or are you currently applying for any patents relating to the content of the manuscript?

- Have you received reimbursements, fees, funding, or salary from an organization that holds or has applied for patents relating to the content of the manuscript?
- Do you have any other financial competing interests?
- Do you have any non-financial competing interests in relation to this paper?

If you can answer no to all of the above, write 'I declare that I have no competing interests' below. If your reply is yes to any, please give details below.

I declare that I have no competing interests

I agree to the open peer review policy of the journal. I understand that my name will be included on my report to the authors and, if the manuscript is accepted for publication, my named report including any attachments I upload will be posted on the website along with the authors' responses. I agree for my report to be made available under an Open Access Creative Commons CC-BY license (http://creativecommons.org/licenses/by/4.0/). I understand that any comments which I do not wish to be included in my named report can be included as confidential comments to the editors, which will not be published.

I agree to the open peer review policy of the journal

To further support our reviewers, we have joined with Publons, where you can gain additional credit to further highlight your hard work (see: https://publons.com/journal/530/gigascience). On publication of this paper, your review will be automatically added to Publons, you can then choose whether or not to claim your Publons credit. I understand this statement.

Yes Choose an item.
