# Supplementary material for: New de novo assembly of the Atlantic bottlenose dolphin (Tursiops truncatus) improves genome completeness and provides haplotype phasing
Source: Gigascience. 2019 Jan 29;8(3):giy168. doi: 10.1093/gigascience/giy168 (PMC6443575; doi:10.1093/gigascience/giy168)
Supplement: Reviewer_2_Report_Original_Submission -- Shengfeng Huang — 8/24/2018 Reviewed [file giy168_reviewer_2_report_original_submission.pdf]

Reviewer Report

Title: New de novo assembly of the Atlantic bottlenose dolphin (Tursiops truncatus) improves genome completeness and provides haplotype phasing

Version: Original Submission **Date:** 8/24/2018

Reviewer name: Shengfeng Huang

Reviewer Comments to Author:

This manuscript provide new genome assemblies for Atlantic bottlenose dolphin based on new sequencing data set. Here are my comments:
1) It appears that the new assemblies did not leverage the old but valuable sequencing data based on Sanger and 454 reads, which together give about ~6X coverage of data. Such data might benefit the contiguity of the assemlby a lot. And this is probably already reflected on the contig N50 size, which is only 30Kb in the new assembly (even hundreds coverage of reads were used), shorter than the previous assemblies based on the 454/sanger reads (37Kb). The authors need to discuss on this.
2) It is not very easy to get the detailed information on what software were used on scaffolding, gap filling, phasing and scaffold splitting/merging. Did the autors used a home-made software, or publicly available tools? Where to access these tools? what parameters were used?
3) The names of Tur_tru_Illumina_hap_v1 and Tur_tru_Illumina_dip_v1 could be somewhat misleading: hap=haploid whereas the dip=diploid. May be haploid versus phased-haplotypes.
4) The synteny analysis appears show no new finding or improvement over the old assembly (which may be due to the fact the old ones are already chromosome-level assembly?). If there is new findings/improvements, the authors are encouraged to show them.
5) There could be more comparative analysis between the new and old assemblies. It appears they are based on different data set. For example, some Venn figures could to use to show the differences between them. LIke, 1000 genes, 900 are both complete in new and old assemblies, while new assemblies has 60 more complete genes and the old have 40 more complete genes but they are not overlaped. And some examples of the genes could be provided.

Level of Interest

Please indicate how interesting you found the manuscript: Choose an item.

Quality of Written English

Please indicate the quality of language in the manuscript: Choose an item.

Declaration of Competing Interests

Please complete a declaration of competing interests, considering the following questions:

- Have you in the past five years received reimbursements, fees, funding, or salary from an organisation that may in any way gain or lose financially from the publication of this manuscript, either now or in the future?
- Do you hold any stocks or shares in an organisation that may in any way gain or lose financially from the publication of this manuscript, either now or in the future?
- Do you hold or are you currently applying for any patents relating to the content of the manuscript?
- Have you received reimbursements, fees, funding, or salary from an organization that holds or has applied for patents relating to the content of the manuscript?
- Do you have any other financial competing interests?
- Do you have any non-financial competing interests in relation to this paper?

If you can answer no to all of the above, write 'I declare that I have no competing interests' below. If your reply is yes to any, please give details below.

I declare that I have no competing interests.

I agree to the open peer review policy of the journal. I understand that my name will be included on my report to the authors and, if the manuscript is accepted for publication, my named report including any attachments I upload will be posted on the website along with the authors' responses. I agree for my report to be made available under an Open Access Creative Commons CC-BY license (http://creativecommons.org/licenses/by/4.0/). I understand that any comments which I do not wish to be included in my named report can be included as confidential comments to the editors, which will not be published.

I agree to the open peer review policy of the journal

To further support our reviewers, we have joined with Publons, where you can gain additional credit to further highlight your hard work (see: https://publons.com/journal/530/gigascience). On publication of this paper, your review will be automatically added to Publons, you can then choose whether or not to claim your Publons credit. I understand this statement.

Yes Choose an item.
